# The GPI sidechain of *Toxoplasma gondii* inhibits parasite pathogenesis

**DOI:** 10.1128/mbio.00527-24

**Published:** 2024-09-20

**Authors:** Julia A. Alvarez, Elisabet Gas-Pascual, Sahil Malhi, Juan C. Sánchez-Arcila, Ferdinand Ngale Njume, Hanke van der Wel, Yanlin Zhao, Laura García-López, Gabriella Ceron, Jasmine Posada, Scott P. Souza, George S. Yap, Christopher M. West, Kirk D. C. Jensen

**Affiliations:** 1 Department of Molecular and Cell Biology, School of Natural Sciences, University of California, Merced, California, USA; 2 Quantitative and Systems Biology Graduate Program, University of California, Merced, California, USA; 3 Department of Biochemistry and Molecular Biology, Center for Tropical and Emerging Global Diseases, and Complex Carbohydrate Research Center, University of Georgia, Athens, Georgia, USA; 4 Department of Medicine and Center for Immunity and Inflammation, New Jersey Medical School, Rutgers University, Newark, New Jersey, USA; 5 Health Sciences Research Institute, University of California, Merced, California, USA; Albert Einstein College of Medicine, Bronx, New York, USA

**Keywords:** GPI, GIPL, GPI sidechain, mass spectrometry, PIGJ, PIGE, surface antigens, macrophages, galectin-3, CD36, pathogenesis, *Toxoplasma gondii*

## Abstract

Glycosylphosphatidylinositols (GPIs) are highly conserved anchors for eukaryotic cell surface proteins. The apicomplexan parasite, *Toxoplasma gondii*, is a widespread intracellular parasite of warm-blooded animals whose plasma membrane is covered with GPI-anchored proteins, and free GPIs called GIPLs. While the glycan portion is conserved, species differ in sidechains added to the triple mannose core. The functional significance of the Glcα1,4GalNAcβ1- sidechain reported in *Toxoplasma gondii* has remained largely unknown without understanding its biosynthesis. Here we identify and disrupt two glycosyltransferase genes and confirm their respective roles by serology and mass spectrometry. Parasites lacking the sidechain on account of deletion of the first glycosyltransferase, PIGJ, exhibit increased virulence during primary and secondary infections, suggesting it is an important pathogenesis factor. Cytokine responses, antibody recognition of GPI-anchored SAGs, and complement binding to PIGJ mutants are intact. By contrast, the scavenger receptor CD36 shows enhanced binding to PIGJ mutants, potentially explaining a subtle tropism for macrophages detected early in infection. Galectin-3, which binds GIPLs, exhibits an enhancement of binding to PIGJ mutants, and the protection of galectin-3 knockout mice from lethality suggests that Δ*pigj* parasite virulence in this context is sidechain dependent. Parasite numbers are not affected by Δ*pigj* early in the infection in wild-type mice, suggesting a breakdown of tolerance. However, increased tissue cysts in the brains of mice infected with Δ*pigj* parasites indicate an advantage over wild-type strains. Thus, the GPI sidechain of *T. gondii* plays a crucial and diverse role in regulating disease outcomes in the infected host.

**IMPORTANCE:**

The functional significance of sidechain modifications to the glycosylphosphatidylinositol (GPI) anchor in parasites has yet to be determined because the glycosyltransferases responsible for these modifications have not been identified. Here we present identification and characterization of both *Toxoplasmsa gondii* GPI sidechain-modifying glycosyltransferases. Removal of the glycosyltransferase that adds the first GalNAc to the sidechain results in parasites without a sidechain on the GPI, and increased host susceptibility to infection. Loss of the second glycosyltransferase results in a sidechain with GalNAc alone, and no glucose added, and has negligible effect on disease outcomes. This indicates GPI sidechains are fundamental to host-parasite interactions.

## INTRODUCTION

Protozoan parasites are widespread and cause prominent diseases including malaria, leishmaniasis, Chagas disease, and toxoplasmosis. One notable feature of protozoans is their extensive decoration of glycosylinositolphospholipids (GIPL) and glycosylphosphatidylinositol-anchored proteins (GPI-AP). The GPI was first discovered and characterized in trypanosomes, the protozoan parasite that causes African sleeping sickness in humans (1). Since its discovery, it has been shown to have a conserved core structure across the eukaryotic kingdom; EtNP-6Manα1-2Manα1-6Manα1-4GlcN1-6*myo*-inositol-phospholipid (where EtNP, Man, and GlcN are ethanolamine phosphate, mannose, and glucosamine, respectively) (1, 2). Apicomplexan parasites use GPI-APs to assist attachment to and cellular invasion of host cells (3). As such, the core GPI synthetic pathway enzymes are each essential gene product required for *Toxoplasma gondii* (*T. gondii*) and *Plasmodium sp*. survival and intracellular infection (4, 5).

In the case of *T. gondii*, a widespread apicomplexan parasite of warm-blooded animals and humans, its cell surface is covered by a large family of GPI-APs belonging to the surface antigen glycoprotein (SAG)-related superfamily (SRS) (6, 7). SAG antigens are targeted by *T. gondii*-specific antibodies following infection (8) and play important roles in virulence. For example, SAG1, the dominant antigen in the parasite’s lytic stage (9), promotes small intestinal ileitis in mice (10) and parasite survival *in vivo* (11) and in activated macrophages, by an unknown mechanism (12). Another GPI-AP, SRS35 (also known as p18 or SAG4), has been shown to promote mouse macrophage invasion and virulence of the parasite (13). By contrast, overexpression of the GPI-AP SRS29C (p35) quells *T. gondii* virulence and promotes mouse survival during an otherwise lethal infection (6).

Many GPI-APs have been considered for their roles in parasite virulence, and studies of the GPI that anchor them have found them to be immunogenic in *T. gondii* and other parasites. For instance, *T. cruzi* GIPLs are potent activators of TLR2 (14). Similarly, GIPLs of *P. falciparum* are thought to be major pathogenesis factors and are shown to activate TLR2 and TLR4 (15), which, in turn, causes lethal inflammatory responses (16). The GIPL of *T. gondii* activates TLR2 and TLR4 (17), and evidence suggests it is recognized by the galactose-binding lectin, galectin-3 (18). Galectin-3 regulates the immune system (19), including inflammatory responses during *T. gondii* infection (20). *T. gondii* and *Plasmodium sp*. GIPLs are also robustly targeted by antibodies during the early stages of infection in both humans and mice (21–29). Collectively, these data demonstrate that parasite GPIs elicit robust innate and adaptive immune responses during infection.

Whereas the core GPI structure is conserved across the eukaryotic kingdom, species differ in how their GPI is modified through the attachment of additional sugars and other moieties to the mannose core, called sidechain modifications (30–32) (Fig. S1). The significance of these sidechains and why species possess structural diversity in their GPI modifications is unclear because the responsible GPI glycosyltransferases have remained unidentified in most species. However, in humans, the glycosyltransferase PGAP4 was recently identified as responsible for linking the β-d-GalNAc of its sidechain disaccharide sidechain to the 4-position of first core Man residue (linked to GlcNH_2_) (33). PGAP4 is widely conserved among eukaryotic species that are similarly substituted, including *Caenorhabditis elegans*. In *T. gondii,* the GPI sidechain occurs on the same first mannose in two glycoforms: one consisting of a single identically linked β-d-GalNAc, and the other in which the GalNAc is extended by an α4-linked d-Glc (21). Interestingly, however, a homolog of PGAP4 is not detected in *T. gondii* despite having the identically linked sugar (Fig. S1) (33). Deletion of PGAP4 mice resulted in elevated blood alkaline phosphatase levels, impaired bone formation, decreased locomotion, impaired memory, and enhanced vulnerability to prion diseases (34). These results point to GPI sidechains having far-reaching and important roles in mammals.

Even less is known regarding the functional significance of GPI sidechains in eukaryotic microbes. For example, in the fungus *Candida albicans*, a fourth mannose is added as a sidechain to the third mannose of the core by the *SMP3* glycosyltransferase. Removal of *SMP3* interferes with fungal viability *in vitro* (35). The agent for malaria, *Plasmodium falciparum* also has a fourth mannose GPI sidechain (Fig. S1), but lacks a homolog to *SMP3*. Interestingly, its *PIGB* GT, which is responsible for adding the third mannose to its GPI core, can also add the fourth mannose when complemented into *C. albicans* Δ*smp3* mutants. However, whether its *PIGB* also mediates this function in *P. falciparum* is uncertain because the addition of the third Man is essential for parasite viability (36). The agent for African sleeping sickness, *Trypanosoma brucei*, exhibits an elaborate array of branched sidechains emanating from the second mannose of its GPI core, especially in its procyclic form. While the GT genes responsible for most of the linkages remain to be mapped and may be impeded by the complexity and redundancy, the deletion of three of the genes encoding GTs that contribute to peripheral linkages is minimally consequential for fitness and virulence (37). Thus understanding the roles of specific glycoforms of GPIs of eukaryotic microbes will require further investigations.

While several eukaryotic GPI sidechain-modifying glycosyltransferases have been identified to date, none have addressed whether the complete absence of a GPI sidechain is required for microbial pathogenicity. We sought to address this question in *T. gondii* and furthermore test whether its GPI sidechain glycoforms impact parasite virulence and immune recognition. Here we identify its two sidechain-modifying GTs and report that loss of the GPI sidechain promotes virulence. Whereas antibody recognition of parasite GPI-APs and inflammatory cytokines appear intact to GPI sidechain null parasites, we present indirect evidence for galectin-3 and the scavenger receptor, CD36, in mediating specific phenotypes associated with sidechain deficiency in *T. gondii* strains.

## MATERIALS AND METHODS

### Parasite strains, cell lines, oligonucleotides, and plasmids

Human foreskin fibroblasts (HFFs) monolayers were grown in DMEM GlutaMAX (4.5 g/L D-glucose) (Life Technologies #10-566-024) supplemented with 2 mM L-glutamine, 20% heat-inactivated (HI) fetal bovine serum (FBS) (Omega Scientific), 1% penicillin-streptomycin (Life Technologies #15140122), and 0.2% gentamycin (Life Technologies #15-710-072). Mouse embryonic fibroblasts (MEFs) were grown in DMEM GlutaMAX (4.5 g/L D-glucose) supplemented with 10% HI FBS, 20 mM HEPES, 1% penicillin-streptomycin, and 0.2% gentamycin. *Toxoplasma gondii* strains were passaged in HFFs in “Toxo medium” (4.5 g/L D-glucose, L-glutamine in DMEM GlutaMAX supplemented with 1% HI FBS, and 1% penicillin-streptomycin). The following clonal strains were used (clonal types are indicated in parentheses): RH Δ*ku80* Δ*hxgprt* (type I), RH *GFP:cLUC* (type I) (clone 1-1), RH Δ*ku80* Δ*hxgprt* Δ*207750::HXGPRT* (type I) (clone C12), RH Δ*ku80* Δ*hxgprt* Δ266320::DHFR-TS* (type I) (38), GT1 (type I), GT1 Δ207750::DHFR-TS* (type I) (clone B8), GT1 Δ266320::DHFR-TS* (type I) (clone C11), CEP *hxgprt*− (type III), CEP *hxgprt− GFP:cLUC* (type III), CEP *hxgprt−* Δ*207750::HXGPRT GFP:cLUC* (type III) (clone D10), CEP *hxgprt−* Δ207750::HXGPRT 207750_3×HA_:DHFR-TS* GFP:cLUC (type III) (clone C2), and CEP *hxgprt−* Δ*266320::HXGPRT GFP:cLUC* (type III) (clone D4). All strains, oligos, and plasmids used to generate strains in this study are shown in Table S1.

### Gene editing and complementation

Cas9 and single-guide RNA (gRNA) expression plasmid, pSS013 (gift from Jeroen Saeij, University of California, Davis) was designed to target the first exon of *Tg_207750* (Uniport S8FC68). For *Tg_266320* (Uniprot S7VW57)*,* a modified dual-guide pU6-universal plasmid targeting two different exons within the gene was previously generated (38). An amplicon was generated by PCR of either the pyrimethamine selectable cassette (DHFR-TS*) from the pLoxp-DHFR-TS-mCherry plasmid (Addgene plasmid #70147) or *HXGPRT* from the pTKO-att plasmid (a gift from Jeroen Saeij, University of California, Davis). When editing the RH Δ*ku80* Δ*hxgprt* strain, homology arms flanking the protospacer sequence were introduced into the *HXGPRT* selectable cassette by PCR. The Cas9/gRNA expression plasmid and amplicon were co-transfected via electroporation at a 5:1 ratio. Transfectants were selected and cloned in Toxo medium containing either 1 µM pyrimethamine or 50 µg/mL of MPA (Axxora) and 50 µg/mL of xanthine (Alfa Aesar). Insertion and orientation of the selectable markers into the Cas9 cut-sites were confirmed by diagnostic PCR (Fig. S2 and S3).

The pLIC-DHFR-TS-3xHA plasmid (gift from Jeroen Saeij, University of California, Davis) was treated with PacI (NEB #R0547S) and AvrII (NEB #R0174S) to allow for insertion of a *TgVEG_207750* complementation construct by homology-directed ligation. The full-length coding region with introns spliced out of *TgVEG_207750* was amplified using the Q5 high fidelity polymerase (NEB #M0491) from a CEP cDNA library using primers designed to contain 19 bp homologous with the 3′ end of the *TgVEG_207750* promoter sequence and 25 bp homologous with end of the digested pLIC-HA plasmid that contains and provides a C-terminal 3× HA tag. The CEP cDNA library was prepared from a CEP total RNA preparation using the High-Capacity cDNA Reverse Transcription Kit (Thermo Fisher #4368814) according to the manufacturer’s instructions. To amplify the *TgVEG_207750* promoter region, a 1,000 bp non-coding region upstream of the start ATG was amplified from CEP genomic DNA with Q5 high fidelity polymerase according to the manufacturer’s protocol. The forward primer contained a 19 bp homology sequence to the end of the digested pLIC-HA plasmid and the reverse shared a 22 bp homology sequence with the 5′ end of the amplified *TgVEG_207750* coding-cDNA sequence described above. The amplified promoter and coding *207750* DNA sequence was first purified from 1% agarose gels using the Zymoclean Gel DNA recovery kit (Zymo Research #D4007) and assembled in frame into the digested pLIC-DHFR-TS-3xHA plasmid with the 3× HA tag using the NEBuilder HiFi DNA Assembly cloning Kit (NEB #E5520) according to the manufacturer’s instructions. The CEP *hxgprt−* Δ*207750::HXGPRT GFP:cLUC* strain was transfected with the linearized pLIC-DHFR-TS 207750_3xHA_ plasmid, grown in pyrimethamine selection medium and cloned by limiting dilution to generate the *CEP* Δ*pigj + PIGJ_3×HA_* complementation strain.

### Fluorescence microscopy

HA-tagged PIGJ was localized by fluorescent microscopy. HFFs (8 × 10^5^) were plated on coverslips in 24-well plates overnight before being infected with 8 × 10^4^ parasites. Cells were fixed with 3% formaldehyde (Polysciences Methanol free, Ultrapure #18814-20) in phosphate-buffered saline (PBS; pH 7.4) (Life Technologies #10010023) and permeabilized with blocking buffer (1× PBS with 5% normal goat serum, 3% bovine serum albumin (BSA) fraction V (Omega FB-11), 0.2% saponin) and stained with rat anti-HA (1:50) (3F10, Sigma). Secondary goat anti-rat AF594 (Thermo Fisher #A11007) antibodies were used at 1:3,000, and DAPI (1:10,000), for detection and visualized using a fluorescent microscope (Nikon Eclipse T*i*-S). For co-localization studies, coverslips were stained as above and additionally incubated with endoplasmic reticulum (ER) demarcating guinea pig anti-SERCA (39) or mouse anti-PDI antibodies (1:200) (provided by Katherine Moen and Silvia Moreno, University of Georgia, in preparation) and detected with goat anti-mouse A647 (Thermo Fisher #A21236) or goat anti-guinea pig A647 (Thermo Fisher #A21450). Confocal microscopy was performed using an LSM 880 microscope (Zeiss), and images were processed with Zeiss Zen software (vs3.9).

### Mice

C57BL/6J (B6), A/J, and *Lgals3*−/− (B6.Cg-*Lgals3^tm1Poi^/*J) mice were purchased from Jackson Laboratories. *Tlr2/4* double knockout B6 mice (B6.129P2*-Tlr4^tm1Aki^* B6.129P2*-Tlr2^tm1Aki^*) were a generous gift from Dr. Greg Barton (UC Berkeley). Mice were maintained under specific pathogen-free conditions at UC Merced. Female mice aged 6–10 weeks were used for experiments unless otherwise stated.

### Parasite infections and serotyping

Parasite injections were prepared by scraping T-25 flasks containing vacuolated HFFs and sequential syringe lysis first through a 25G needle followed by a 27G needle. The parasites were spun at 34 × *g* for 5 minutes to remove debris and the supernatant was transferred, followed by a spin at 611 × *g* and washing with sterile 1× PBS (Life Technologies, #10-010-049). For primary infections, mice were infected intraperitoneally (i.p.) with 10^4^ tachyzoites of parental and mutant strains. The parasite viability of the inoculum was determined by a plaque assay. In brief, 100 or 300 tachyzoites were plated in HFF monolayers grown in a 24-well plate and 4–6 days later were counted by microscopy (4× objective) (Nikon Eclipse T*i*-S).

At 30–35 days after primary infection, 50 µL of blood was harvested and collected in Eppendorf tubes containing 5 µL 0.5 M EDTA and placed on ice. Blood was pelleted at 9,391 × *g* for 5 minutes, and blood plasma was collected from the supernatant and stored at −80°C. To evaluate the seropositivity of the mice, HFFs were grown on coverslips and infected with a green fluorescent protein (GFP)-expressing RH (1-1) overnight. 18 hours later, cells were fixed with 3% formaldehyde in PBS, permeabilized with a permeabilization solution (3% BSA fraction V [Omega, FB-11], 2% normal goat serum [Omega, NG-11], 0.2% Triton X-100, 0.01% sodium azide), incubated with a 1:100 dilution of collected blood plasma for 2 hours at room temperature, washed with 1× PBS, and detected with Alexa Fluor 594-labeled goat secondary antibodies specific for mouse IgG (Thermo Fisher #A11032) in permeabilization solution. Seropositive parasites were observed by immunofluorescence microscopy.

For secondary infections, seropositive mice previously infected with CEP *hxgprt*− were infected i.p. with 5 × 10^4^ tachyzoites of type I RH Δ*ku80* parental and mutant strains. Parasite viability of the inoculum was determined by a plaque assay as described above.

### Cyst enumeration using Dolichos-FITC

To quantify brain cysts, brains were dissected and placed in 10 mL of 1× PBS on ice. Brains were homogenized using a 10-mL syringe with an 18G needle by extrusion several times through the needle. The homogenate was spun at 611 × *g* for 7 minutes and then resuspended in 1 mL of 1× PBS. 100 µL of the brain homogenate was fixed in ice-cold methanol at a 1:10 dilution for 5 minutes, spun in a microcentrifuge at 5,200 × *g* for 5 minutes and washed once and resuspended in 1 mL 1× PBS. The fixed homogenate was stained with a 1:150 dilution of FITC-conjugated Dolichos biflorus agglutinin (Vector Laboratories #FL-1031) in 1× PBS and slowly rotated at 4°C overnight. Samples were washed twice with 1× PBS and resuspended in 1 mL of 1× PBS. Stained homogenate (50 µL) was plated in a 96-well plate and four replicates of each sample were enumerated for cysts with an inverted fluorescence microscope using a 20× objective (Nikon Eclipse T*i*-S).

### Superinfections

Mice that survived 35 days of secondary infection were euthanized and brain homogenates were generated and resuspended in 1 mL of 1× PBS as described above. Homogenate (100 µL) was cultured in HFFs and monitored for parasite growth. Once parasite growth was established, the media was switched to selection media containing MPA/xanthine for secondary infections to select against the primary infecting CEP *hxgprt−* strain, and for *HXGPRT* expressing secondary infection strain. Parasites that grew in selection media were also confirmed to be type I strains through restriction fragment length polymorphism analysis. In brief, isolated DNA was PCR amplified using GRA6 primers, and the purified amplicon was fragmented with MseI (NEB #R0525M). Products were run on a gel to determine parasite type I or III based on unique fragmentation sizes indicative of the genotype.

### SDS-PAGE and immunoblotting for parasite lysate antigen

To generate parasite lysate antigens, *T. gondii* was cultured in HFF monolayers and expanded to approximately 2 × 10^7^ parasites in a T175 flask. Parasites were syringe lysed, washed with sterile 1× PBS, and pelleted at 611 × *g* for 7 minutes. The pelleted parasites were lysed with Laemmli buffer (0.0625 M Tris-HCl, 0.07 M SDS, 10% glycerol, 5% β-mercaptoethanol, pH 6.8) or 0.1% Triton-X100 in PBS and centrifuged at 14,000 × *g* for 20 minutes to remove large insoluble debris. The supernatant was aliquoted and stored at −80°C. Parasite lysates were separated via SDS-PAGE in 4%–20% acrylamide hand cast gels before transfer to the PVDF membrane. Membranes were blocked with 10% fortified bovine milk (Raley’s) dissolved in Tris-buffered saline (TBS) (0.05 M Tris-HCl, 0.15 M NaCl, pH 7.4) with 0.1% Tween (TBS-T 0.1%) for 1–2 hours at room temperature or overnight at 4°C. Blots were then probed with *T. gondii* GPI glycoform-specific antibody clones (1:1,000) T3 3F12 (BEI) which binds GalNAc GIPL, or T5 4E10 (a gift from Jean Francois Dubremetz U. Montpelier), which is reported to have enhanced affinity for GalNAc + Glc GIPL of *T. gondii* (21) but can bind GalNAc GPI intermediates of humans (33) in blocking buffer for 72 hours at 4°C. For loading controls, blots were probed (1:2,000) with anti-SAG1 (T4 IE5, BEI). Membranes were washed with TBS-T 0.1% three times and incubated for 1 hour at room temperature with goat anti-mouse Ig horseradish peroxidase (HRP)-conjugated antibodies (Southern Biotech, anti-IgM secondary 1:1,000 [#1020-05], anti-IgG 1:7,500 [#1030-05]). Membranes were washed with TBS-T 0.1% three times and developed with Immobilon Forte Western HRP Substrate (Millipore WBLUF0500). All blots were imaged via chemiluminescence on a ChemiDoc Touch (Bio-Rad #12003153). Image Lab 6.1 software (Bio-Rad) was used for the analysis of bands.

### NanoLC-MS and MALDI-TOF-MS analysis of GIPLs and GPI-anchored protein glycans released from parasites

Parasite pellets corresponding to approximately 3 × 10^8^ parasites were harvested as described in above but from 15 T175 flasks and thoroughly rinsed in ice-cold PBS (Corning cat # 21-040 CV), before being pelleted and stored at −80°C. Control, uninfected HFF monolayers were harvested in 1 mM EDTA in PBS and washed three times in ice-cold PBS by centrifugation (8 minutes, 2,000 × *g*, 4°C). Parasite and host cell pellets were then delipidated, and the protein pellet was extracted with butan-1-ol saturated water to enrich GPI-anchored proteins, as described previously (38). GIPL fractions were obtained from the clarified lipid extracts generated during initial pellet delipidation. Fatty acids were removed from both GPI-anchored proteins and GIPLs by incubation in 0.5 M NH_4_OH (in water for GPI-anchored proteins or after bath sonication in 70% ethanol for GIPLs) under rotation for 6 hours at 4°C. The glycan core was then released by hydrofluoric acid treatment, and C18 was cleaned up, re-N-acetylated, permethylated, and dried as described previously (38).

For nLC-MS, permethylated glycans were redissolved in MeOH and 10 µL was mixed with 90 µL 0.1% formic acid in water. 10 µL was injected into a PepMap Acclaim analytical C18 (75 µm, 15 cm, 2 µm pore size) column maintained at 60°C in an Ultimate 3000 RSLC coupled to a Q-Exactive-Plus mass spectrometer (Thermo Fisher Scientific). The column was equilibrated for 10 minutes at 97.5% LC-MS buffer A (0.1% formic acid in water) and ramped up to 35% LC-MS buffer B (80% [vol/vol]) over 2 minutes. Glycan separation was achieved using a linear gradient from 35% to 70% buffer B over 150 minutes at a flow rate of 300 nL/min. The effluent was introduced into the mass spectrometer by nanospray ionization in positive mode via a stainless-steel emitter with spray voltage set to 1.9 kV and capillary temperature set at 275°C. The MS method consisted of a survey Full MS scan at 70,000 resolution in positive ion mode, followed by MS(2) fragmentation of the top 10 most intense peaks using HCD at 40% collision energy and an isolation window of 2 *m*/*z*. Dynamic exclusion was set to exclude ions for fragmentation for 30 s. All the data were processed manually using the Xcalibur 2.0 software package.

For MALDI-TOF-MS, permethylated samples were redissolved in MeOH and 0.5 µL aliquots were mixed with 0.5 µL MeOH saturated with either 2,5-dihydroxybenzoic acid (DHB) or α-cyano-4-hydroxycinnamic acid (CHCA) on the MALDI plate and allowed to air dry. Samples were then analyzed on an ABI 4700 MALDI-TOF-MS operated in positive ion reflectron mode for *m*/*z* range 500–5,000.

### Flow cytometry of peritoneal exudate cells after infection

Mice were infected i.p. with 10^6^ tachyzoites, and 3 hours post-injection mice were euthanized and peritoneal cavity lavaged with 4 mL of 1× PBS and 3 mL of air. The peritoneal exudate fluid was passed through a 70-µm cell strainer, cells were pelleted for staining. Samples were blocked for 30 minutes in FACS buffer (2% FBS 1× PBS) containing Fc Block anti-CD16/32 (2.4G2, BD Biosciences) (1:100 dilution), 5% normal hamster serum, and 5% normal rat serum (Jackson ImmunoResearch). After blocking, cells were stained at a 1:100 dilution for 30 minutes with the following antibodies: CD11b BUV395 (M1/70, BD), Gr.1 PE (RB6-8C5, BioLegend), F4.80 BV421 (BM8, Thermo Fisher), MHC II APC (AF6-120.2, Thermo Fisher), B220 APC-Cy7 (RA3-6B2, BioLegend), CD19 BUV785 (6D5, BioLegend), NK 1.1 PE Cy7 (PK136, Thermo Fisher), CD3ε BV510 (17A2, BD), and propidium iodine (PI) (1:1.000). After incubation, cells were washed three times in FACS buffer and resuspended in FACS buffer. Samples were run on a ZE5 flow cytometer (Bio-Rad) and analyzed using FlowJo software version 10.

### Flow cytometry for surface antigens

Syringe-lysed parasites were fixed in 3% formaldehyde in PBS for 20 minutes, washed in PBS, plated in a 96-well micro-titer plate at 8 × 10^5^ parasites per well, and incubated with one of the following antibodies at (1:100) to detect antigen expression: mouse anti-SAG1 (T4 IE5, BEI), mouse anti-SAG3 (T4 1F12, BEI), and mouse anti-p35 (T4 3F12, BEI). After 20 minutes of incubation on ice, parasites were washed twice with FACS buffer and stained with (1:100) goat anti-mouse IgG-APC (BioLegend #Poly4053). After incubation, cells were washed and resuspended in FACS buffer. Samples were run on a ZE5 flow cytometer and analyzed using FlowJo software version 10. Staining was assessed on forward and side scatter characteristics consistent with parasites and further gated for GFP+ if the parasite expressed GFP.

For GIPL surface staining, syringe-lysed and fixed parasites were stained on ice in FACS buffer for 30 minutes with T3 3F12 (1:400) and T5 4E10 (1:100), and anti-SAG3 (T4 1F12, BEI) to mark parasites. Parasites were washed and stained (1:100) with detection antibodies anti-IgG3-BV421 (R40-82, BD Bioscience), anti-IgM-PE/Cy7 (RMM-1, BioLegend), and anti-IgG2a-PerCP/Cy5.5 (RMG2a-62, BioLegend) for 30 minutes. After incubation, cells were washed and resuspended in FACS buffer. Samples were run on a ZE5 flow cytometer and analyzed using FlowJo software version 10. Staining was assessed on forward and side scatter characteristics consistent with parasites and further gated for SAG3+ parasites.

### Serum antibody parasite binding assay

For serum reactivity analysis, syringe-lysed parasites were fixed in 3% formaldehyde for 20 minutes, washed twice in PBS, and plated in 96-well microtiter plates at 4 × 10^5^ parasites/well. The parasites were then incubated with serum from chronically infected mice, in concentrations ranging from 10^−2^ to 10^−6^ diluted in FACS buffer, for 20 minutes at 37°C. Parasites were then washed with FACS buffer and placed on ice for incubation with anti-isotype detection antibodies depending on application: anti-IgG3-BV421 (R40-82, BD Bioscience), anti-IgM-PE/Cy7 (RMM-1, BioLegend), anti-IgG1-APC (RMG1-1, BioLegend), anti-IgG2b-PE (RMG2b-1, BioLegend), and anti-IgG2a-PerCP/Cy5.5 (RMG2a-62, BioLegend). Parasites were washed and resuspended in FACS buffer as described before. Flow cytometry was performed on an LSR analyzer (BD) and analyzed using FlowJo software version 10. The MFI was computed by gating on forward and side scatter characteristics consistent with parasites and further gated for GFP+ if the parasite expressed GFP using FlowJo.

### Complement C3b-binding assay

For analysis of C3b binding, parasites were syringe-lysed, washed, and resuspended to a concertation of 1 × 10^7^ parasites per mL in Hanks’ balancing salt solution buffer (HBSS) (Thermo Fisher #14175095). Parasites were plated in a 96-well plate at 10^6^ parasites per well with 10% blood plasma from naïve mice and incubated at 37°C for 30 minutes. After incubation, complement activation was stopped by washing with cold 1× PBS. Parasites were spun down at 611 × *g* for 3 minutes and washed three times using cold 1× PBS. Parasites were then fixed using 3% formaldehyde for 10 minutes and then washed using cold 1× PBS. Parasites were then stained with mouse anti-C3b (10C7) (Invitrogen MA1-70054) (1:200) for 20 minutes. After primary stain, parasites were washed with cold 1× PBS and stained with anti-mouse IgG1 APC (RMG1-1, BioLegend) (1:100) for 20 minutes on ice. After incubation, parasites were washed three times and resuspended in 1× PBS for analysis. Samples were run on a ZE5 machine, and data were analyzed using FlowJo version 10.3. MFI was determined as described above.

### Galectin-3 binding assay

Recombinant Gal-3 (R&D Systems #1197-GA-050) was pre-diluted in PBS containing 10% BSA and 14 mM β-mercaptoethanol. Parasites were syringe-lysed and washed, and 8 × 10^6^ parasites were incubated with either 10 or 5 µg of recombinant Gal-3 for 1 hour at 4°C in FACS buffer. Parasites were washed to remove unbound Gal-3 and incubated with a PE-conjugated anti-Gal-3 antibody (eBioM3/38 [M3/38], Invitrogen) at a 1:100 dilution in FACS buffer for 20 minutes at 4°C. After two further washes, the samples were analyzed using a ZE5 Cell Analyzer (Bio-Rad), and data were processed as described above.

### CD36-Fc binding assay

Parasites (10^7^) were incubated with 0.5 µg recombinant human IgG Fc (R&D Systems # 110-HG) or recombinant CD36-Fc (R&D Systems, #2519 CD) in 50 µL binding buffer (0.14 M NaCl, 2.5 mM CaCl_2_, 0.01 M HEPES [pH 7.4], 3% BSA) for 1 hour at 15°C. Parasites were washed once to remove unbound recombinant protein and the parasites were then incubated with mouse anti-SAG1-AF405 (TP3cc, Novus Biologicals) and donkey anti-human IgG-Daylight 550 (Thermo Fisher, SA5-10127). The stained parasites were acquired by Attune NxT flow cytometry. The CD36-Fc MFI was computed by gating on GFP+/SAG1+parasites using FlowJo.

### Opsonization and invasion assays

Bone marrow-derived macrophages (BMDMs) were generated in L929 conditioned medium from female C57BL/6J mice as previously described (40). BMDMs were plated a day in advance on a coverslip lined 24-well plate at 8 × 10^5^ cells in 1 mL per well, in “BMDM media” supplemented with 10% L929 (20% HI FBS, 1% penicillin-streptomycin, 1% non-essential amino acids [Thermo Fisher #11-140-050], 1% sodium pyruvate). On the day of the experiment, freshly lysed parasites were washed and resuspended for an MOI of 0.5 in Toxo medium warmed to 37°C. In a 96-well plate, serum dilutions were made in pre-warmed Toxo medium. Parasites were added to the diluted serum and incubated at 37°C for 20 minutes before being added to the BMDM coverslip wells with fresh Toxo medium. The BMDM plates were spun at 304 × *g* for 5 minutes to synchronize parasite invasion between wells and incubated at 37°C for 40 minutes to allow for phagocytosis or invasion. After 40 minutes, media was replaced with 300 µL of 3% formaldehyde in 1× PBS, fixed for 20 minutes, washed three times with 1× PBS, blocked and permeabilized with “Blocking Buffer” for 1 hour (1× PBS with 10% normal goat serum, and 0.1% saponin). Cells were stained with rabbit anti-GRA7 (1:5000) (gift from John Boothroyd, Stanford) and rat anti-LAMP1 (1D4B, BD Biosciences) (1:500) diluted in blocking buffer for 1 hour or overnight at 4°C. Wells were then washed three times with 1× PBS before secondary antibody incubation in blocking buffer with goat anti-rabbit AF647 (1:3,000) (Thermo Fisher #A21245), goat anti-rat AF594 (1:1,000) (Thermo Fisher #A11007), DAPI (1:10,000), and as indicated, goat anti-Toxo conjugated FITC (1:400) (ViroStat #0283). Fluorescence microscopy was performed (Nikon Eclipse T*i*-S) and images were captured at 60× and blinded for analysis. Images were processed (Nikon Elements) and 80–100 GFP+ or FITC+ events were quantified for parasite association with Lamp1+ or for containment within a ring-like GRA7+ parasitophorous vacuole (PV); fraction opsonization was calculated as (Lamp1+GFP+ counts/Lamp1+GFP+ plus GRA7 PV+GFP+ total counts).

For invasion assays, BMDMs were plated on coverslips in 24-well plates overnight as described above and infected the next day at a multiplicity of infection (MOI) of 0.25 parasites. Plates were spun at 304 × *g* to synchronize invasion and incubated at 37°C for 20 minutes before washing once in 1× PBS and fixing in 3% formaldehyde. Cells were not permeabilized. Wells were washed and stained with mouse anti-SAG1 (1:5,000) for 1 hour. Cells were washed, and secondary antibodies goat anti-mouse AF594 (1:3000) and DAPI (1:1,000) were used for 1 hour. Fluorescence microscopy was performed as described above, with 100 GFP+ parasites counted per slide and quantified for parasites for SAG1+ staining. Invaded parasites were determined to be GFP+ SAG1−; the fraction of invasion was calculated as (SAG1−GFP+ counts/total GFP+ counts).

### Neutralization assay

MEFs were plated a day in advance in a 96-well plate at 5 × 10^5^ cells per well in MEF media. On the day of the experiment, freshly lysed parasites were washed and resuspended to a concentration of 1.6 × 10^7^ in Toxo medium warmed to 37°C. In a 96-well plate, blood plasma 1:10 dilution was made in pre-warmed Toxo medium and incubated at 37°C in 10% serum for 20 minutes before being added to the MEFs, such that in the absence of serum the MOI is approximately 0.5. The plate was spun at 304 ×*g* for 3 minutes to synchronize parasite invasion and incubated at 37°C for 2 hours. The supernatant was carefully removed, and 20 µL of trypsin was added to each well and incubated at 37°C for 5 minutes to dislodge cells from the plate. Cells were harvested with 100 µL of cold 1× PBS, transferred to a FACS plate, and washed three times with FACS buffer to remove trypsin. Cells were resuspended in 1:1,000 PI in FACS buffer and then analyzed via flow cytometry for GFP+ PI– MEFs to indicate invasion. Neutralization was defined as a ratio: the percentage of PI– (viable) cells infected with parasites incubated with serum divided by the percentage of PI– cells infected with parasites without serum incubation. For non-GFP+ expressing parasites, cells were fixed after trypsinization in 3% formaldehyde, washed, and blocked for 1 hour before staining with anti-Toxo-FITC (ViroStat #0283). Neutralization ratios were determined as described, but PI was not included in the discrimination. Samples were run on a ZE5 machine, and data were analyzed using FlowJo software version 10.

### Parasite lytic cycle

For plaque sizes, HFFs were plated in 24-well plates and allowed to grow to confluency before adding 100 or 300 syringe-lysed parasites and allowed to grow for 5 days. Plaque sizes were measured (Nikon Elements) under 4× objective (Nikon Eclipse T*i*-S).

For parasites per vacuole assays, 8 × 10^5^ MEFs were plated on coverslips in 24-well plates and allowed to adhere before infecting with 10^5^ parasites. Plates were spun at 475 × *g* to synchronize invasion and incubated for 15 minutes at 37°C. After 15 minutes, cells were washed twice to remove any unattached parasites. Fresh Toxo media was added, and plates were incubated at 37°C for 16 hours before being fixed, blocked with 0.1% saponin blocking buffer (as described above), and stained for 1 hour with rabbit anti-GRA7 (1:3,000). Cells were washed and incubated with secondary goat anti-rabbit AF 594 (1:3,000) (Thermo Fisher #A11037) and DAPI (1:10,000) for 1 hour in 0.1% saponin blocking buffer. 100 GFP+ vacuoles per slide were quantified for a number of parasites residing in GRA7+ vacuoles under 100× objective (Nikon Eclipse T*i*-S).

For attachment time course assays, HFFs were plated in 24-well plates and allowed to grow to confluency. 200 syringe-lysed parasites were added per well and plates were spun at 475 × *g* to synchronize invasion and incubated for 30, 60, or 90 minutes before washing five times in 1× PBS to remove any unattached parasites. Fresh Toxo medium was added and plates were incubated at 37°C for 5 days before counting plaques under the microscope. Plaque numbers were normalized to those obtained from wells that did not undergo any washes.

### Quantitative PCR for cytokine expression and parasite burden

Cells were collected from mouse peritoneal lavages as described above and spleens. Cells were filtered through a 70-µm filter and centrifuged 611 × *g* for 7 minutes to pellet and washed with sterile 1× PBS before pelleting again. Spleenocyte samples were removed of red blood cells through treatment of ACK lysis buffer (0.5 M NH_4_Cl, 0.01 M KHCO_3_, 0.0001 M EDTA) for 5 minutes before being washed with 1× PBS. Cells were stored in RNA-later (Thermo Fisher #AM7021) at −80°C until RNA isolation. After thawing samples, aliquots were removed and diluted with 1× PBS and centrifuged to pellet cells and removed RNA-later. RNA was isolated using the RNEasy Mini Kit (QIAGEN #74134) and cDNA was synthesized using the Invitrogen High-Capacity cDNA Reverse Transcription Kit (Thermo Fisher #4368814). Quantitative PCR was performed on synthesized cDNA using iTaq Universal SYBR Green Supermix (BioRad #172511). Normalization of all samples was calculated in comparison to *Actb* expression levels. Fold change in cytokines was determined through the 2^–ΔΔCt^ method.

For parasite burden determination by quantitative PCR, peritoneal exudate and spleen cells, liver, and lung tissues were harvested, and appropriate amounts were digested according to the manufacturer’s protocols using the QIAGEN DNeasy Blood & Tissue Kit (#69504). Brains were isolated and DNA purified as follows. In brief, whole brains were harvested in 5 mL of 1X PBS and homogenized through an 18G needle of a 10 mL syringe and passed up and down through the needle 10 times before being spun down at 611 × *g* for 7 minutes to pellet. 100 µL of brain homogenates was processed using the QIAGEN DNeasy Blood & Tissue kit according to the manufacturer’s directions. Standard curves were made from known numbers of isolated parasite DNA ranging from 10^1^ to 10^6^ parasites per well. Isolated DNA samples were amplified targeting the *Toxoplasma-*specific B1 gene, and the standard curve was used to quantify parasite number in each sample per μg of isolated DNA.

### Statistics

Statistical analyses were performed with GraphPad Prism 8 software. Statistical significance was defined as *P* < 0.05. *P* values between the two groups were calculated using paired or non-paired two-tailed t-tests. One- or two-way ANOVA was used for comparison across more than two groups with Tukey or Dunnett correction, as recommended by Prism. Fisher’s exact *t*-test was used to assess categorical differences in superinfection. Survival curve significance was calculated using log-rank Mantel-Cox testing.

## RESULTS

### *Tg_207750* and *Tg_266320* are predicted to be the *T. gondii* GPI sidechain-modifying glycosyltransferases PIGJ and PIGE, respectively

**Fig 1:**
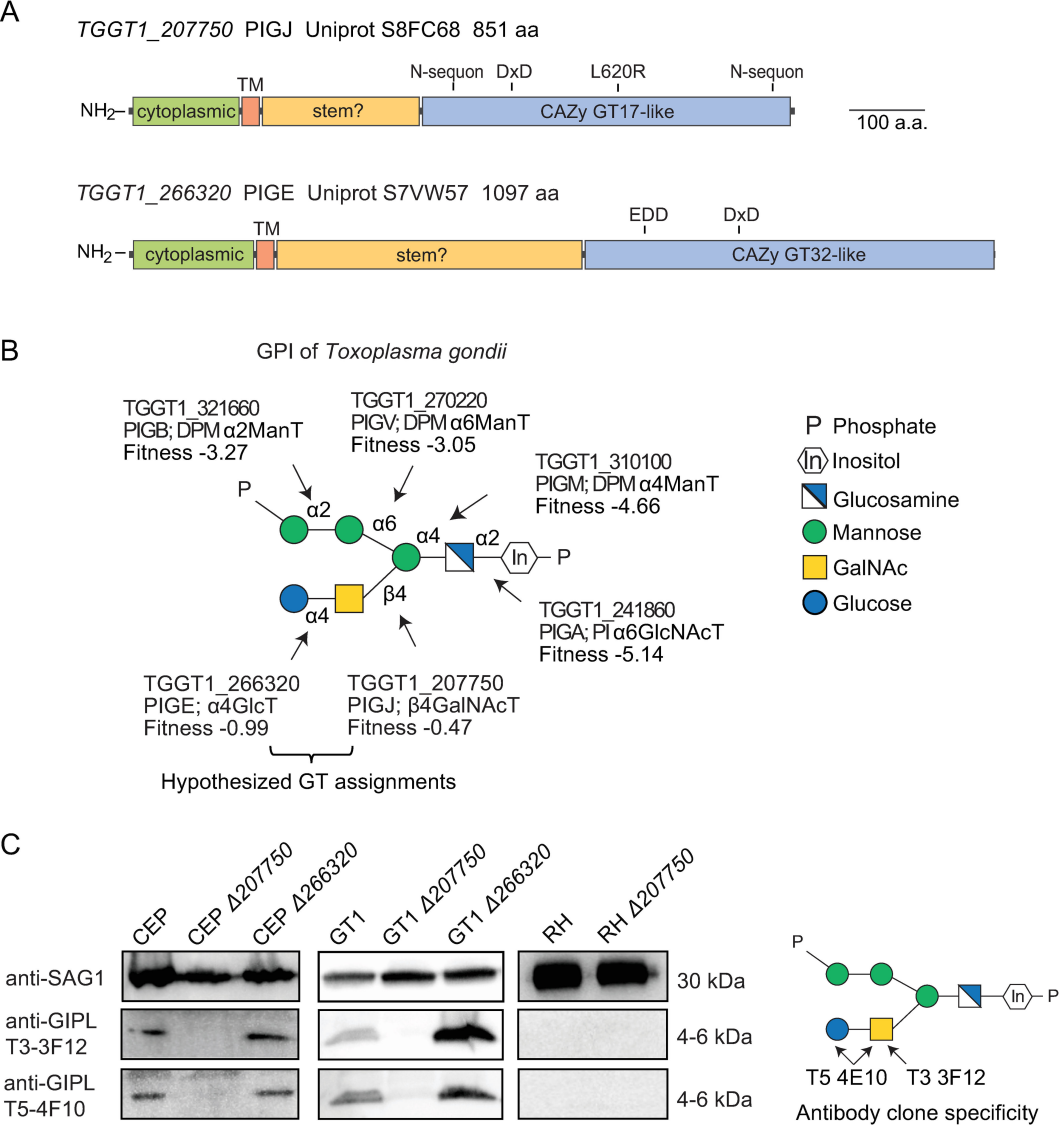
Glycoform-specific antibodies implicate *Tg_207750* as the GalNAc GPI sidechain-modifying enzyme, PIGJ. (**A**) Protein models for *Tg_207750* (PIGJ) and *Tg_266320* (PIGE) from ToxoDB were used to seed BLASTp and other homology searches to predict the evolution and function of the proteins. Regions of predicted location or function are labeled: cytoplasmic, transmembrane (TM), stem, a CAZy GT domain, DxD (active site motif), EDD (DxD-like motif), N-sequon (for N-glycan), and a non-synonymous SNP between type I strains. (**B**) Schematic of the *T. gondii* GPI with known and putative glycosyltransferases (GTs) involved in the GPI synthetic pathway. Labels show their gene ID, gene name, transferase type, fitness score (the more negative the fitness score being more lethal to the parasite when inactivated) (4), and the linkage they form. Two novel GTs hypothesized to perform the indicated transferase activity are named “PIGJ” and “PIGE.” (**C**) Representative western blots confirming the loss of the sidechain in Δ*207750* mutants through GPI sidechain-specific antibody reactivity to the low molecular weight (4–6 kDa) GIPL antigen. Schematic indicating the GIPL glycoform specificity of antibody clones T3 3F12 (binds the GalNAc glycoform) and T5 4E10 (binds both GalNAc +/− Glc glycoforms). RH GIPL sidechains were neither detected by T3 3F12 nor T5 4E10. Loading controls were performed using anti-SAG1 (T4 IE5). Representative of 4–6 experiments.

*T. gondii* strains are known to differ in virulence in various intermediate hosts (41). In laboratory mice, type III strains are referred to as avirulent because they require a dose of over 10^5^ parasites to achieve 50% lethality (41, 42). Meanwhile, type I strains are highly virulent, and one parasite is enough to be 100% lethal in naïve mice (43, 44). However, when C57BL/6J mice are given a primary infection with the avirulent type III strain CEP and allowed to progress to a chronic infection, they survive secondary infections (i.e., “challenge”) with high-dose type I strain RH parasites due to protective immunological memory responses induced by the initial parasite exposure (45–47). By contrast, when mice infected with CEP are challenged with another type I strain, GT1, they succumb (46). RH and GT1 are highly similar, but the gene *Tg_207750* was noted to be six times more highly expressed in RH compared to GT1 and predicted to be a glycosyltransferase that adds an N-acetylated hexosamine (HexNAc) to a core mannose of complex N-glycans (48). Informatic analysis suggests it encodes a type 2 transmembrane protein (49) with a C-terminal inverting glycosyltransferase domain with greatest similarity to CAZy family GT17 (38) (Fig. 1A). The putative catalytic DxD motif is separated from the transmembrane domain by a stretch of about 170 aa, which in other GTs comprises a stem-like region that may mediate protein interactions or appropriately orient the catalytic domain. The 143-aa putative cytoplasmic region is predicted to be disordered based on amino acid composition. AlphaFold predictions confirm the potential of the GT17 region to fold as a GT-A superfamily domain but do not confidently predict a structure for the stem-like or cytoplasmic regions (not shown). *Tg_207750* was one of two *T. gondii* βHexNAc transferase-like GTs we proposed to be responsible for adding the GalNAc to the GPI sidechain of *T. gondii* (38). The GT responsible for GalNAc sidechain addition, we name here as “PIGJ” (Fig. 1B), we hypothesized to be *Tg_207750* and might be partially responsible for differences in secondary infection virulence between RH and GT1 type I strains.

In addition, the gene *Tg_266320* was previously suggested to encode the GPI αGlc GT that mediates the addition of the terminal Glc to GalNAc to complete the disaccharide sidechain (38). The GT responsible for this activity we name here as “PIGE” (Fig. 1B). *Tg_266320* is also predicted to be a type 2 transmembrane protein, with a CAZy GT32 retaining glycosyltransferase domain (38) separated from the transmembrane domain by a ~385 aa long stem-like region, and a ~160 aa poorly conserved and disordered putative cytoplasmic region (Fig. 1A). GT32 GTs utilize sugar nucleotide rather than Dol-P-sugar donors, as previously documented for the enzyme that assembles the Glc α1,4-linkage in the *Toxoplasma* GPI (50) and, consistent with this activity, encodes a DxD motif in its GT domain. We tested the hypothesis that *Tg_266320* encodes the α4GlcT or PIGE.

### Western blot analysis implicates *Tg_207750* as PIGJ

To address their roles in the GPI sidechain synthetic pathway, each predicted GT gene was targeted for disruption by CRISPR-Cas9 and repair with a drug-selectable marker (Fig. S2 and S3). Mutants were made in both type I (RH, GT1) and type III (CEP) genetic backgrounds (Table S1), and integration of the selectable markers was confirmed with diagnostic primers and PCR (Fig. S2 and S3). Using glycoform-specific antibodies that preferentially bind the GalNAc form (T3 3F12) or both sidechain glycoforms (T5 4F10) (21, 33), Western blot analysis revealed these antibodies lose recognition of GIPLs from the GT1 Δ*207750* and CEP Δ*207750* strains (Fig. 1C). This indicates that the enzyme encoded by *Tg_207750* is likely the PIGJ transferase responsible for GalNAc addition to the mannose core of the GPI. Western blot analysis of GT1 Δ*266320* and CEP Δ*266320* mutants retained GIPL recognition by the T3 3F12 and T5 4F10 antibodies (Fig. 1C). Though *Tg_207750* is more highly expressed in RH compared to GT1 type I strains (48, 51), we do not detect GIPL in the RH strain using these antibodies (Fig. 1C), nor did we detect the sidechain by mass spectrometry of GPI anchors isolated from this strain (38). These findings support the identity of *Tg_207750* as encoding the GalNAc transferase but leave the role of *Tg*_*207750* in the RH background unclear.

The binding potential of the GIPL-specific antibodies to intact parasites was also assessed by flow cytometry (Fig. S4). T3 3F12 staining of the GT1 Δ*207750* mutant was significantly reduced compared to the parental strain, but enhanced to the GT1 Δ*266320* mutant. Comparing type I strains, T3 3F12 staining of the RH strain was reduced compared to GT1 and barely above isotype staining (Fig. S4), results that are in concordance with the Western blot analysis (Fig. 1C). T5 4F10 showed considerable heterogeneity between experiments, but the intensity of staining was relatively low across all strains. Finally, when compared to isotype control staining, anti-GIPL staining of the mutant strains was not entirely abrogated, indicating antibody recognition of epitopes independent of either putative glycosyltransferase. Owing to ambiguous findings using the T5 4F10 antibody, and overall staining results surrounding Δ*207750* and Δ*266320* parasite strains, we turned to direct analysis of GIPLs and GPI anchors using mass spectrometry.

### Structural analysis confirms GPI sidechain glycosyltransferases PIGJ and PIGE of *T. gondii*

**Fig 2:**
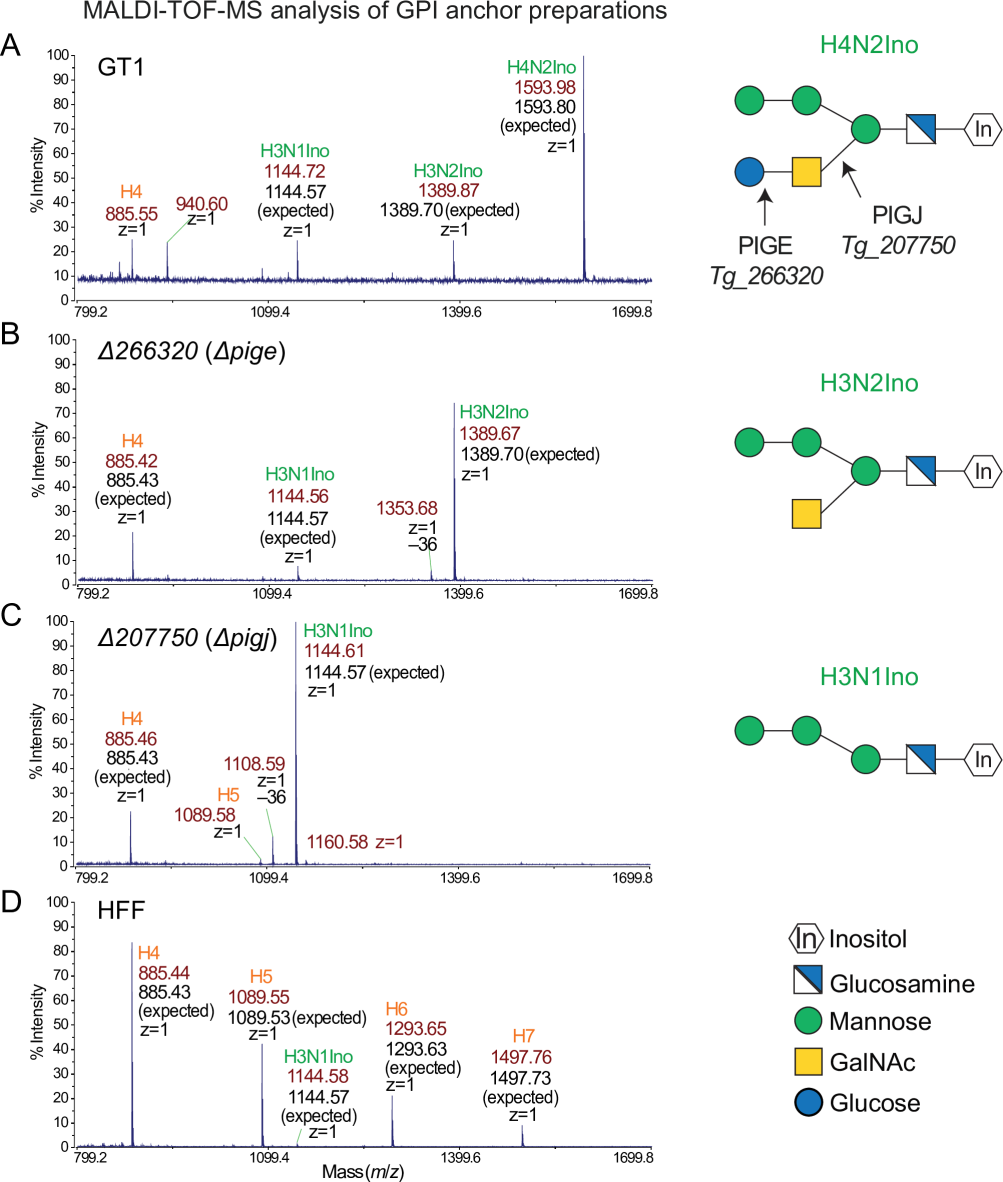
MALDI-TOF-MS analysis of GPI preparations from GPI-anchored proteins reveals the identities of PIGE and PIGJ as GPI sidechain glycosyltransferases. Glycans isolated from GPI-anchored proteins from tachyzoite stage parasites were N-acetylated, permethylated, mixed with CHCA matrix, and analyzed in reflectron and positive ion mode. The *m*/*z* range of 800–1,700 is shown. Ions that correspond to singly charged (*z* = 1) sodiated GPI glycans are labeled in green, with the observed monoisotopic *m*/*z* value (in dark red), and the predicted *m*/*z* values for the assignment (in black). As an example, the composition assignment for an ion possessing three hexoses (known to be mannoses), one HexNAc (known to be N-acetylglucosamine), and one hexitol (known to be inositol) is represented as H3N1Ino. Ions that correspond to Hex(n) oligomers are labeled in orange. (**A**) Parental GT1 strain. (**B**) Δ*pige*. (**C**) Δ*pigj*. (D) Host cells (HFFs). Schematics indicating the *T. gondii* GPI with confirmed GT assignments for PIGJ and PIGE, and corresponding hexose composition of the various glycans are shown.

For an independent assessment of the roles of *Tg_207750* and *Tg_266320*, GIPL and GPI anchors from wild-type and mutant GT1 parasites were subjected to structural analyses (Fig. 2). We previously developed a new method to isolate and analyze the glycan component of GPI anchors using mass spectrometry (38). The method first enriched GPI-anchored proteins using butan-1-ol extraction of material delipidated by extraction with CHCl_3_ and MeOH. The fraction was saponified to remove potential fatty acids, and the glycan core was released by phosphodiester bond cleavage with hydrofluoric acid. Finally, the glycan was N-acetylated and permethylated and analyzed initially by MALDI-TOF-MS. The most abundant ion from the parental GT1 strain corresponded to a glycan with the composition of four hexose residues, two HexNAcs, and one hexitol, or H4N2Ino (Fig. 2A). This composition corresponds to the previously described structure consisting of three Man, one Glc, one GalNAc, and one inositol, as shown in Fig. 1B (21). In addition, less abundant ions were found that correspond to H3N2Ino and H3N1Ino, suggesting the presence of GPI glycoforms with a monosaccharide sidearm, or none at all. Ions whose composition corresponded to multimers of hexose, H3, H4, and H5, were also detected.

Since parasites are grown in HFFs, we assessed the potential contribution of HFFs to these ions, as mammals also express GPI anchor glycans with a disaccharide sidechain on the α4-linked (first) Man residue (33). Preliminary analysis of N-glycans, performed as described (38), showed the presence of N-glycans from HFFs as well as tachyzoites (data not shown), such that >25% of the sample was potentially of HFF origin. Therefore, a GPI-anchor fraction from HFFs was examined. As shown in Fig. 2D, this sample contained a series of hexose oligomers whose abundance decreases with length, which potentially represent breakdown products of glycogen, as well as an ion corresponding to H3N1Ino. Previous analysis of preparation of spontaneously lysed extracellular tachyzoites that were unlikely to be contaminated by host cells, and that were washed extensively to minimize serum contaminants, yielded no detectable hexosamers (38), consistent with the low level of starch accumulation at the tachyzoite stage. However, the ratio of H3N1Ino to Hex4 was small in HFFs compared to the large ratio in the GT1 sample (Fig. 2A and D). If the amount of H4 is used as a measure of contamination of HFFs to the parasite to sample, it is evident that the great majority of the GPI glycans originated from the parasites.

**Fig 3:**
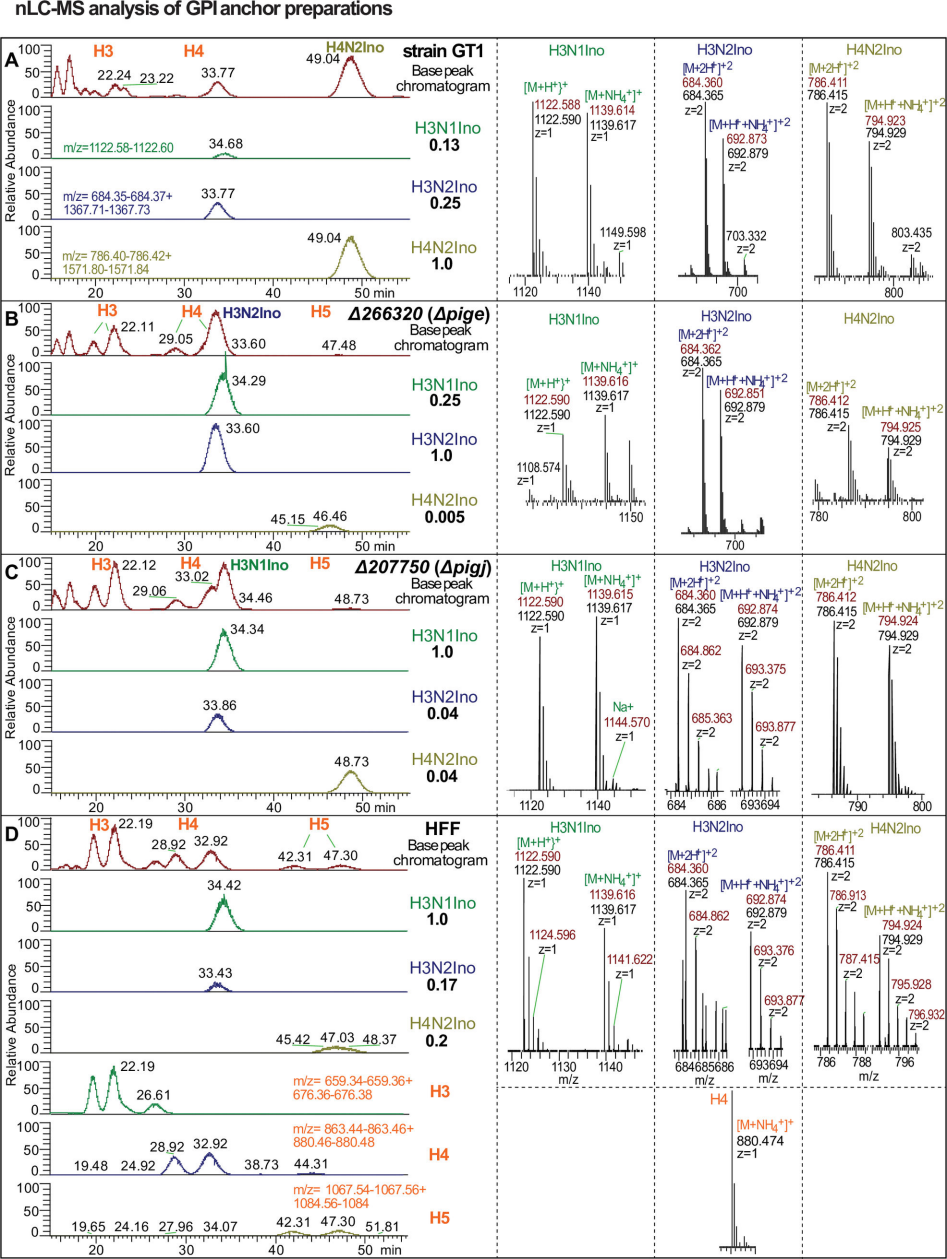
nLC-MS analysis of GPI-anchor preparations confirms the GPI sidechain transferase activity of PIGJ and PIGE in *T. gondii*. Isolated GPI permethylated glycans described in Fig. 2 were reanalyzed by nLC separation on a C18 column and hyphenated analysis in an Orbitrap mass spectrometer in positive ion mode. The left column of panels shows base peak chromatograms (all ions m/z 500–2,000) and extracted ion chromatograms (EIC) for each of the indicated targets (m/z ranges are specified in panels A and D). The ratio of ion intensities over the EIC *m*/*z* ranges shown for H3N1Ino, H3N2Ino, and H4N2Ino is shown relative to the most abundant ion. The most abundant ions are labeled in the base peak chromatogram. The right column of panels shows representative mass spectra of the two most abundant ions eluting with the target and used for quantitating relative levels. Levels of hexosamers were not quantitated. Observed *m*/*z* values are in dark red, expected values are in black, and the charge state is as indicated. (**A**) Parental GT1 strain. (**B**) Δ*pige*. (**C**) Δ*pigj*. (**D**) Host cells (HFFs).

To verify these interpretations, the GT1 GPI preparation was also examined by MS analysis in an Orbitrap mass spectrometer after separation by C18 nano-HPLC. As shown in Fig. 3, the elution profile of all ions (base peak chromatogram) from the GT1 strain included a prominent peak eluting at 49.04 min (Fig. 3A). This peak coincided with the extracted ion chromatogram analysis (EIC) of H4N2Ino in the lower trace of panel A. A second peak eluting at about 33.77 min corresponded to the elution positions of H3N1Ino and H3N2Ino, which were not well resolved by this method. These ions are eluted as a mixture of primarily singly charged H^+^ or NH_4_^+^ ions, and doubly charged ions in either 2H^+^ or H^+^/NH_4_^+^ states, as shown in the right-hand panels. These ions matched the expected *m*/*z* values with 1–2 orders of magnitude higher accuracy compared to the MALDI-TOF-MS method. Furthermore, collision-based decomposition (HCD MS-2) revealed a series of daughter ions that confirmed that it consisted of various combinations of monosaccharides including non-reducing end Hex residues (Fig. S5A) that are completely consistent with the structure shown in Fig. 1B. As expected, all three GPI glycoforms were detected, but at a ratio of 1.0:0.25:0.13 for H4N2Ino:H3N2Ino:H3N1Ino, respectively (Fig. 3A), consistent with the MALDI-TOF-MS data (Fig. 2A). Most of the remaining ions in the base peak chromatogram matched the elution positions of the H3, H4, and H5 hexosamers, as described for the HFF GPI sample (Fig. 3D). Note that these isomers elute in pairs with the same *m*/*z* value, indicating that they represent a mixture of reducing end α- and β-anomers with distinct elution times. While all three GPI isoforms were also detected in the HFF samples, they were of very low relative abundance and H3N1Ino rather than H4N2Ino was the most abundant ion. Taken altogether, the results confirm the presence of the previously described Glc-GalNAc- sidechain of parasite GPI anchors, and good evidence for isomers with only GalNAc or no sidechain at all. The results indicate that GalNAc addition occurs subsequent to the assembly of the tri-mannose core in *T. gondii*.

To test whether the *Tg_266320* gene product is the predicted PIGE GPI Glc transferase, the GPI fraction from Δ*266320* parasites was analyzed as described above. Strikingly, only the H3N2Ino and H3N1Ino isoforms were detected, along with substantial levels of H4 and H5 (Fig. 2B). Using the more sensitive nLC-MS method, the full-length H4N2Ino isoform was detected but at 0.005% of the level of H3N2Ino (Fig. 3B), which is likely a contaminant from the HFF cells. To confirm the predicted role of the *Tg_207750* gene product as the PIGJ GPI GalNAc transferase, the GPI fraction from Δ*207750* parasites, whose disruption of exon 1 (Fig. S2) is expected to interrupt protein expression, was similarly analyzed. In this case, essentially only the H3N1Ino glycan was detected together with substantial H3 and H4 glycans (Fig. 3C). The trace levels of H4N2Ino and H3N2Ino glycans detected were readily explained as carryover from the host HFF cells as indicated by the abundant H3 and H4 glycans. Both mutant forms of the GPI-anchor fragmented to yield expected glycan products (Fig. S5B and C).

*T. gondii* GIPLs were also investigated. The Schwarz group identified the same GPI sidechain glycoforms in both SAG1 (52) and GIPLs (21), and thus we predicted that the same enzymes are responsible for the GIPL sidearm assembly. To address this, the method for isolating the GIPL glycan core was applied to the organic fraction obtained from the initial delipidation step. Using MALDI-TOF-MS, but with a different matrix, a series of ions was observed that suggested the presence of H4N2Ino, H3N2Ino, H3N1Ino, and H4 (Fig. S6A). However, the *m*/*z* values for the parasite glycans were 36 units smaller than expected, whereas H4 had the expected value. In comparison, a parallel analysis using a different matrix yielded the expected *m*/*z* for the most abundant ion (Fig. S6A inset) albeit also a substantial −36 ion. In addition, ions at −14 units were detected at low abundance, suggesting the absence of one methylation event. To examine whether these mass defects were an artifact of MALDI-ionization, the sample was also analyzed by nLC-MS. Only ions corresponding to the expected values were detected (Fig. S7A), and ions with mass defects of −36 and −14 were negligible. Therefore, these defects, which were also present at trace levels in the GPI-anchor analysis (Fig. 2) but not originally noted, were evidently artifacts of the MALDI ionization method. The ratio of H4N2Ino:H3N2INo:H3N1Ino (1.0:0.33:0.17) was like that of the GPI isoform ratios observed in the MALDI-TOF-MS method.

Analysis of the GIPL fractions from Δ*266320* and Δ*207750* strains yielded the same findings as from the GPI-anchor structural analysis. Using either MALDI-TOF-MS (Fig. S6B) or nLC-MS (Fig. S7B), the Δ*266320* sample contained primarily the monosaccharide sidechain with negligible disaccharide sidechain presence. Similarly, the Δ*207750* sample lacked the sidechain altogether (Fig. S6C and S7C) with miniscule levels of sidechain isoforms attributable to host cell contamination (Fig. S6D and S7C). Thus, the same glycosyltransferases act to generate the Glc-GalNAc- side arm in both GPI-APs and GIPLs and a significant fraction of the glycans contain a monosaccharide or no side arm in the wild-type strain as well. In summary, mass spectrometry, together with informatics analyses indicating the presence of GT domains, confirmed the predicted role of both glycosyltransferases. *Tg_266320*, which is required for the addition of the terminal Glc, is hereon referred to as PIGE; *Tg_207750*, which is required for the addition of the GalNAc, is hereon referred to as PIGJ.

### PIGJ mutants cause lethal outcomes during primary and secondary infections

**Fig 4:**
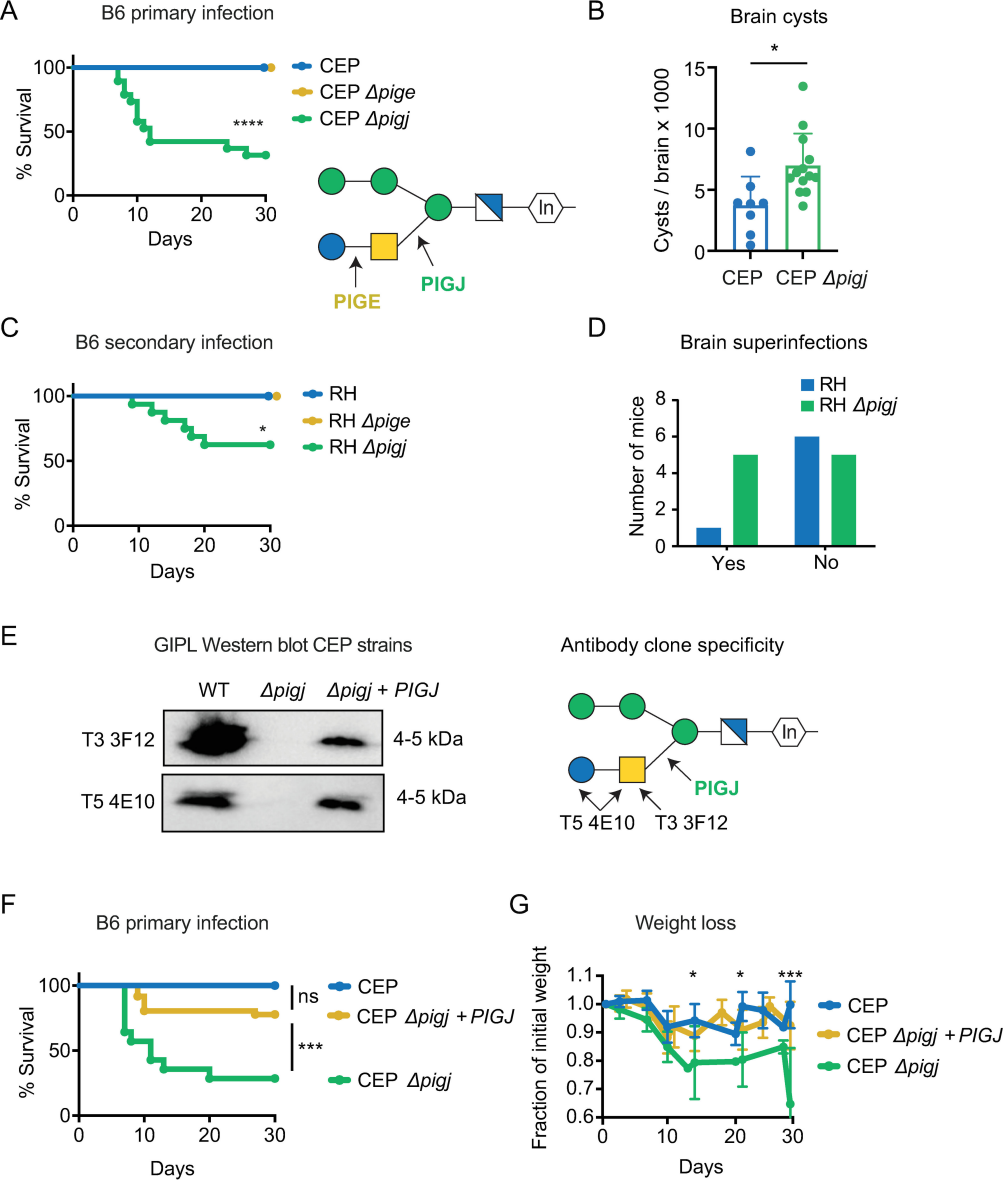
Deletion of the GPI sidechain glycosyltransferase PIGJ but not PIGE results in increased parasite virulence. (**A**) C57BL/6J (B6) mice were given primary infections of 10^4^ parasites (i.p.) of the indicated variants of the type III CEP *hxgprt- GFP:cLUC* strains. Cumulative survival from 2 to 4 experiments is plotted (mice; *n* = 20 CEP, *n* = 19 CEP Δ*pigj*, *n* = 11 CEP Δ*pige*). Schematic represents the *T. gondii* GPI with transferase activities of PIGJ and PIGE. Significance was determined by log-rank (Mantel-Cox) test, ****P < 0.0001. (**B**) Brain cysts were numerated from survivors of the indicated strain. Each dot represents an individual mouse, and the cumulative average + SD from two experiments is plotted. Significance was assessed using an unpaired *t*-test, *P < 0.05. (**C**) B6 mice given a primary infection with CEP *hxgprt*− and 35 days later (i.e., “chronically infected”) were given a secondary infection with 5 × 10^4^ parasites of the indicated variants of the type I RH strain and tracked for survival. Cumulative survival from four experiments is plotted (mice; *n* = 9 RH, *n* = 16 RH Δ*pigj*, *n* = 6 RH Δ*pige*); log-rank (Mantel-Cox), *P < 0.05. (**D**) Brains from survivors in C were homogenized and cultured for parasite growth. After growth was detected, the presence of challenging strain was tested for growth in selection media (MPA Xanthine) and by GRA6 RFLP analysis that can distinguish type I, II, and III lineage strains (*n* = 7 RH, 10 RH Δ*pigj*). Fisher’s exact t-test was performed and the difference was not significant, *P* = 0.3. (**E**) Western blot analysis using GPI glycoform-specific antibodies reveals the GPI sidechain is restored in the CEP Δ*pigj + PIGJ_3×HA_* complementation strain. Schematic indicating the glycoform specificity of antibody clones T3 3F12 and T5 4E10. (**F**) B6 mice were injected i.p. with 10^4^ parasites and monitored for survival for 30 days. Cumulative results from five experiments are plotted (mice; *n* = 4 CEP, *n* = 14 CEP Δ*pigj*, *n* = 36 CEP Δ*pigj + PIGJ*); log-rank (Mantel-Cox), ***P < 0.001, ns, not significant. (**G**) As in F, but weight loss after primary infection was normalized to the initial weight (=1), the average ±SD of each cohort is shown. Statistical significance for weight loss was calculated by one-way ANOVA with multiple comparisons and a Dunnett correction (D14 and D21 *P < 0.05 CEP vs. CEP Δ*pigj*; D28 ***P < 0.001 CEP vs. CEP Δ*pigj*; all other comparisons were not significant, not shown).

With the knowledge that PIGJ and PIGE are the *T. gondii* GPI sidechain GTs, the opportunity presented itself to test the role of distinct GPI glycoforms in microbial virulence. To this end, both primary and secondary infections were performed using the PIGJ and PIGE mutant strains. Importantly, primary infections with the CEP Δ*pigj* strain resulted in 60% mortality compared to 0% mortality in wild-type infections (Fig. 4A). By contrast, mice infected with CEP Δ*pige* survived primary infections (Fig. 4A). During chronic infection, significantly more brain cysts were found in the mice infected with CEP Δ*pigj* compared to wild-type-infected mice (Fig. 4B). Secondary infections were performed in mice chronically infected with the type III strain, and as previously reported (46, 47), these mice survive type I RH challenge due to the formation of protective immunity (Fig. 4C). In this setting, RH Δ*pigj* but not RH Δ*pige* mutants caused lethal secondary infections. There was also increased presence of RH Δ*pigj* in brains of survivors (“superinfection”) compared to RH-challenged survivors, though this difference did not reach statistical significance (Fig. 4D). Chronically infected C57BL/6J mice were also challenged with type I GT1 mutants and succumbed similarly to all three strains: GT1, GT1 Δ*pigj,* and GT1 Δ*pige* (Fig. S8A). Given that C57BL/6J mice are susceptible to GT1 challenges (46), it is difficult to observe virulence increases in this context.

The GPI sidechain mutants were also screened for survival outcomes in A/J mice, which have been shown to exhibit enhanced resistance to primary (53) and secondary infections (54). As observed in C57BL/6J mice, CEP *Δpigj* but not CEP Δ*pige* caused lethality during primary infections in A/J mice (Fig. S8B). During secondary infections with the RH and GT1 GPI sidechain mutants, A/J mice were still resistant (Fig. S8C). Hence, the Δ*pigj* deletion produces a virulence phenotype that overcomes the genetic basis for resistance to primary but not secondary infections in A/J mice. Collectively, these data suggest that the GPI sidechain of *T. gondii* prevents lethal outcomes in its hosts and that host susceptibility is less impacted by the terminal Glc residue transferred by PIGE. PIGJ mutants have an advantage during chronic infection, and the data are consistent with the original hypothesis that differential *Tg_207750* (*PIGJ*) gene expression contributes to the type I strain differences in secondary infection virulence, at least in the susceptible C57BL/6J genetic background.

### PIGJ is expressed in the ER and promotes host survival to *T. gondii*

A PIGJ complementation strain was generated in the CEP Δ*pigj* background and efforts were focused on primary infection and subcellular localization of PIGJ. The full-length coding region with introns spliced out of *TgVEG_207750* was amplified from cDNA and fused with 1,000 bps of the gene’s promoter, followed by delivery as a transgene into CEP Δ*pigj* parasites using a pLIC-DHFR-3xHA plasmid which adds a C-terminal hemagglutinin (HA) tag to the gene of interest (55). Western blot analysis shows the complementation clone, CEP Δ*pigj + PIGJ_3×HA_*, has its GPI sidechain restored (Fig. 4E). Survival and weight loss were also assessed in C57BL/6J mice. Compared to CEP Δ*pigj* infections, a significant increase in mouse survival occurred following primary infection with the complementation strain (Fig. 4F), and when measuring weight loss, the phenotype was complemented to wild-type levels (Fig. 4G).

**Fig 5:**
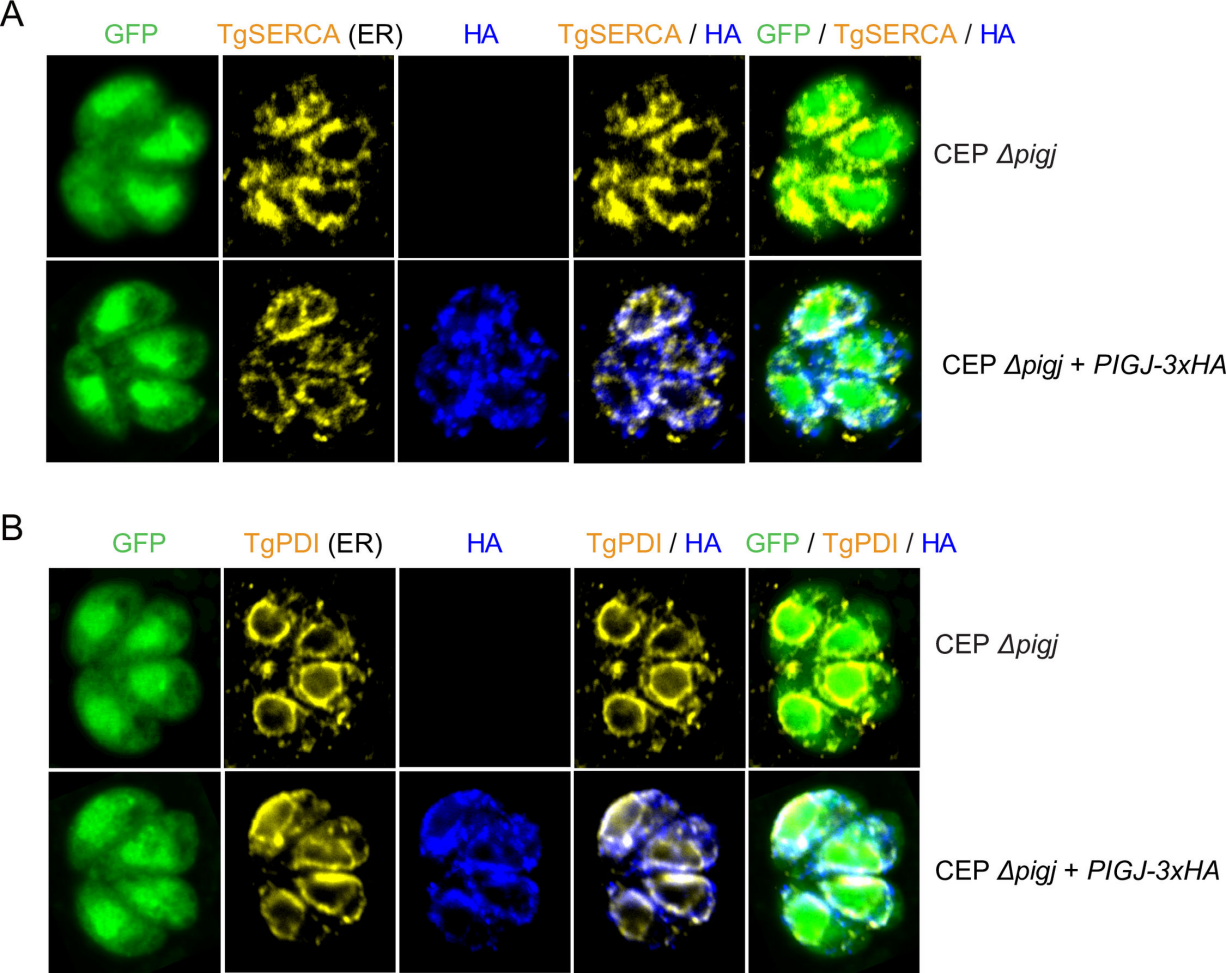
Immunofluorescence analysis of PIGJ-3×HA shows its localization both inside and outside the rER. Confocal fluorescence microscopy of a CEP *hxgprt− GFP:cLUC* Δ*pigj::HXGPRT* parental strain and a derivative expressing *PIGJ_3×HA_*. (**A**) Co-localization of anti-HA (blue) with anti-SERCA (yellow), a marker for parasite rER. Parasites residing within host HFFs are demarcated by GFP. Images are merged as indicated, with overlap between the anti-HA and anti-SERCA signals shown in white. (**B**) Similarly for anti-HA co-localization with anti-PDI, a second marker for the rER.

Confocal microscopy confirmed intracellular expression and localization of PIGJ_3×HA_ (Fig. 5). Anti-HA revealed partial co-localization of PIGJ_3×HA_ with an antiserum against SERCA, which labels the rough endoplasmic reticulum (rER) of intracellular tachyzoites (39, 56) (Fig. 5A). The control strain lacking expressed PIGJ_3×HA_ was negative, confirming antibody specificity. Similarly, anti-HA partially colocalized with an antiserum against PDI (Fig. 5B), a protein enriched in the rER (57) and at the cell surface under certain conditions not present here. The findings are consistent with natural expression of PIGJ_3×HA_ in the rER, which is the site for synthesis of the core GPI structure (58, 59). However, we cannot exclude the possibility that native PIGJ has a broader distribution in the secretory pathway, as PIGJ_3×HA_ localization might be influenced by overexpression or its C-terminal tag.

To assess whether fitness defects may explain the *in vivo* phenotypes, lytic cycle analysis was performed. Consistent with genome wide fitness studies (4) (Fig. 1B), deletion of PIGJ did not impede parasite growth in HFFs or MEFs, nor attachment to host cells (Fig. S9). It was noted CEP Δ*pigj* had significantly larger plaque areas than wild-type CEP, and this gain in plaque size was retained in the complementation strain, suggesting that either the level of complementation of PIGJ was insufficient or the background of the Δ*pigj* strain gained a growth advantage during cloning irrespective of the *PIGJ* gene (Fig. S9). Regardless, differences in *in vitro* growth rates between strains do not correlate with their pathogenesis phenotypes. In summary, host susceptibility inversely correlates with the expression of *PIGJ* and implicates the GPI sidechain of *T. gondii* as a factor that modulates disease outcomes in the infected host.

### Parasite burden and cytokine responses are unchanged following CEP Δ*pigj* primary infections

**Fig 6:**
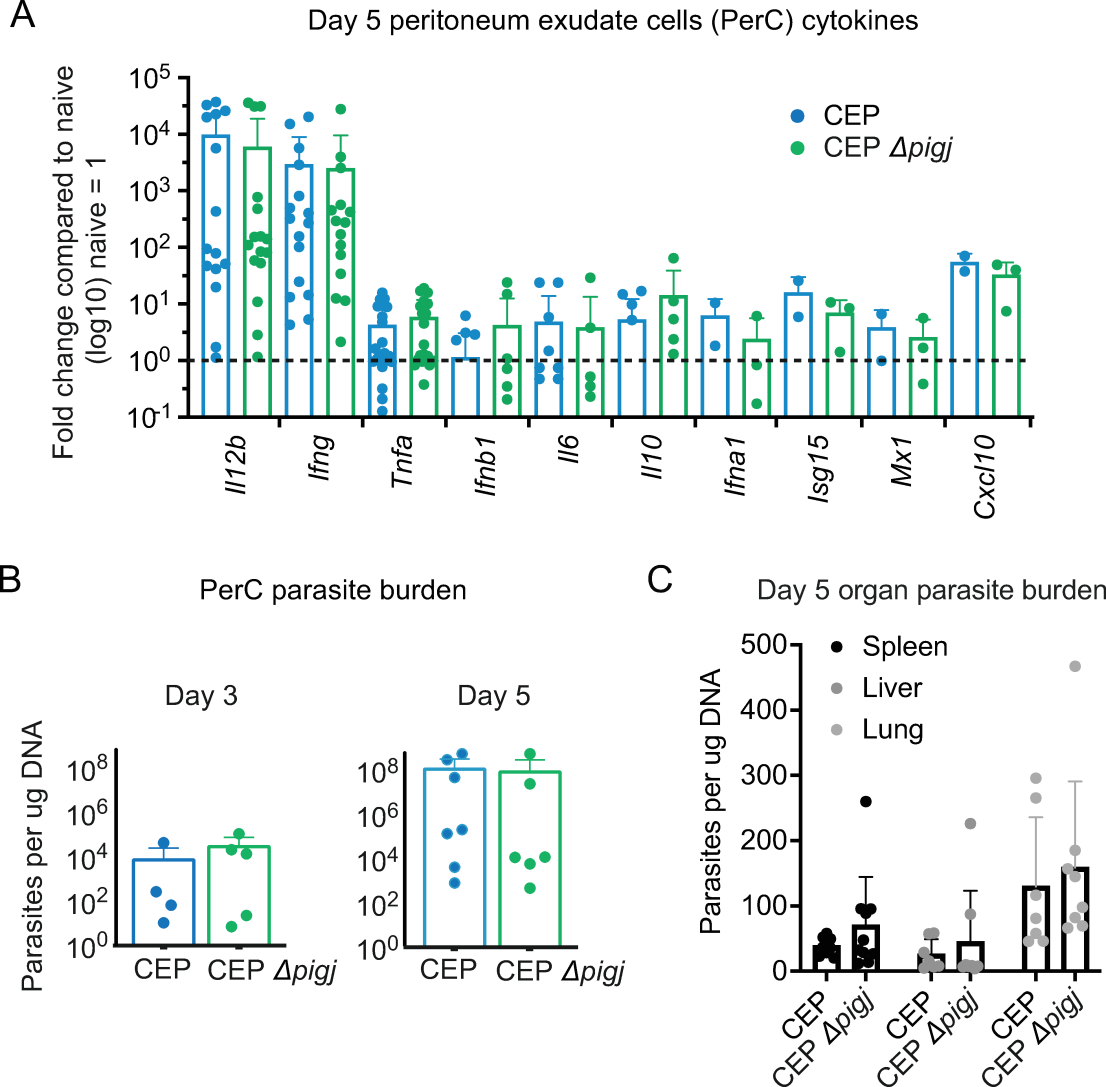
Early parasite burden and cytokine responses are similar between CEP Δ*pigj* and wild-type strains. (**A**) Day 5 after primary infection with the indicated parasite strains in C57BL/6J mice, peritoneal cavity exudate cells (PerC) were harvested and RNA isolated. cDNA was synthesized from RNA and qPCR was performed to measure gene expression levels (fold change), which were normalized to uninfected mouse levels (naïve = 1). Cumulative results from 2 to 5 experiments are plotted. Each dot is the result of an individual mouse. (**B**) Day 3 and (**D**) day 5 after infection, DNA was isolated from PerC, and qPCR was performed using the *Toxoplasma*-specific B1 primers to measure parasite burden relative to the DNA in each sample. Cumulative results of 2–3 experiments are plotted, with each dot representing an individual mouse. (**C**) 5 days after infection, spleen, liver, and lungs were harvested, DNA was isolated, and qPCR was performed using the *Toxoplasma*-specific B1 primers to measure parasite burden between infections. Each dot represents the result of an individual mouse and cumulative results from two experiments are plotted. No differences between CEP and CEP Δ*pigj* infections were significant using an unpaired t-test at *P* < 0.05. See Fig. S10 for additional data.

The early weight loss and death of PIGJ mutants during the first 14 days of primary infection suggested an immune defect. *T. gondii* elicits a robust TH1 response and IFNγ is essential for survival; therefore, gene expression of major cytokines and chemokines known to be involved in TH1 immunity (*Il6, Il12b, Ifng, Cxcl10*) and its regulation (*Il10*) were measured. As GIPL induces robust TLR2/4-dependent TNFα production in macrophages (17), this cytokine was measured (*Tnfa*), as well as type I IFN (*Ifna, Ifnb1*) and type I IFN-induced genes (*Isg15, Mx1*) due to the protective role this pathway plays during primary *T. gondii* infection (60). While there were slight increases in some cytokine responses to infection by the PIGJ mutant compared to wild-type CEP infections, no significant differences were detected in peritoneal exudate cells (PerC) on day 3 (Fig. S10A) or 5 of infection (Fig. 6A), or in splenocytes (Fig. S10B). Moreover, parasite burden in the peritoneum, spleen, lung, and liver appeared the same between mutant and parental strain as measured by qPCR (Fig. 6B and C), or by flow cytometry for GFP+ parasites in the peritoneum (Fig. S10C). Therefore, host susceptibility to primary infection with the PIGJ mutant is not related to overt dysregulation in the cytokine response or parasite burden during acute infection.

### Antibody reactivity, function, complement binding and expression of GPI-anchored SAGs remain largely intact to PIGJ mutants

**Fig 7:**
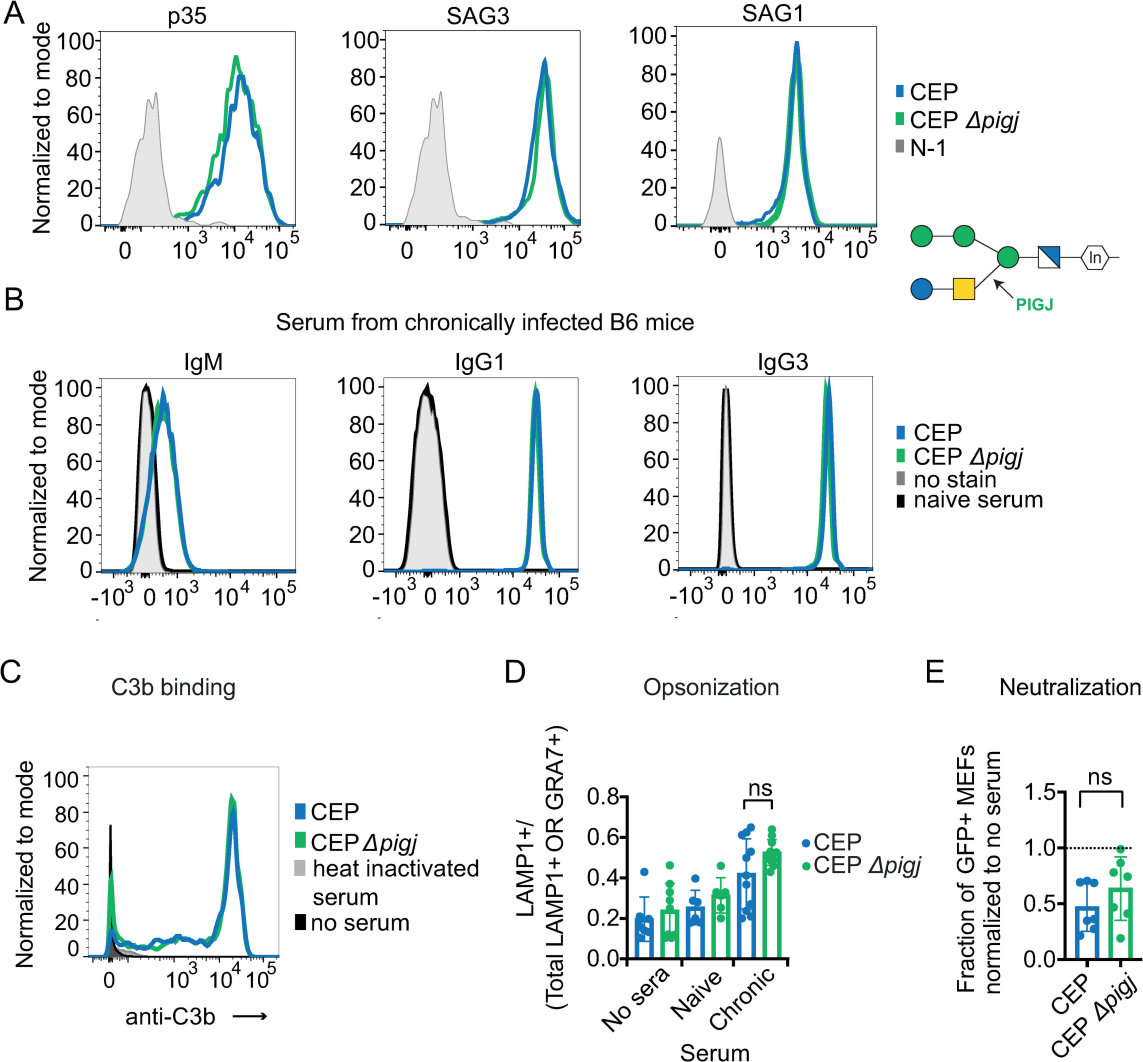
The GPI sidechain does not impact the surface expression of GPI-anchored SAGs, C3b binding, antibody recognition, and functions against *T. gondii*. (**A**) Parasite surface expression of p35, SAG3, and SAG1. Fixed parasites were incubated with primary antibodies against the respective surface antigen, and secondary fluorescent anti-isotype antibodies were used to measure via flow cytometry. Representative histograms of four different experiments shown for CEP and CEP Δ*pigj*. Schematic of the *T. gondii* GPI with the GT activity of PIGJ indicated. (**B**) Fixed parasites were incubated with serum from CEP chronically infected C57BL/6J mice, and antibodies bound to parasites were detected with fluorescent anti-isotype antibodies. Representative histograms of 2–3 experiments displaying parasite-specific antibody reactivity to the indicated CEP strains at 10^−2^ serum dilution. (**C**) Live parasites were incubated with serum from naïve mice and C3b binding was measured with an anti-C3b antibody. Representative histogram from four experiments each with different naïve serums is shown. Staining controls include the use of heat-inactivated serum and N-1, which is the staining in the absence of serum. (**D**) Live parasites were incubated with 1% serum from either CEP chronically infected C57BL/6J mice (“chronic serum”) or naïve mice for 20 minutes before allowing them to invade or be phagocytosed for 40 minutes. Opsonization was calculated as LAMP1+/total LAMP1+ or GRA7+ for each parasite observed. Each dot represents the ratio obtained after counting 100 parasites by fluorescence microscopy for an individual serum or condition, and samples were blinded. Plotted is the average ratio + SD; no sera or naïve serum controls were also assessed. (**E**) Parasites were incubated with 10% chronic serum from C57BL/6J mice for 20 minutes before presentation to MEFs for 2 hours. MEF invasion was quantified for a fraction of GFP+ cells by flow cytometry, and normalized to infections in the absence of serum. Each dot represents the result from an individual serum. Statistics for opsonization was calculated with one-way ANOVA with Tukey correction and for neutralization were calculated with unpaired t-tests; ns, not significant. See Fig. S11 for additional related data.

Evidence indicates that the glycan portion of the GPI can influence the conformation or orientation of the anchored protein (61–63). Therefore, the surface expression and accessibility of major GPI anchored surface proteins SAG1, SAG3, and p35 on wild-type and Δ*pigj* strains were measured with monoclonal antibodies by flow cytometry. For both RH (Fig. S11A) and CEP strains (Fig. 7A), deletion of PIGJ did not prevent surface expression or detection of the aforementioned SAGs. As an alternative approach, the strains were probed with antiserum from chronically infected mice, and fluorescently labeled anti-isotype secondary antibodies were used to detect isotypes of parasite-bound serum antibodies as previously described (54). Again, no differences were observed for IgM, IgG3, IgG1, Ig2a/c, and IgG2b reactivity to wild-type and PIGJ CEP (Fig. 7B) or RH (Fig. S11B) mutants over a range of serum dilutions (not shown). Complement component 3 (C3) binds the surface of *T. gondii* and is required for host resistance to infection (64). Incubating parasites with naïve serum as a source of complement and detection with an anti-C3 antibody revealed no difference in C3 recognition between CEP and Δ*pigj* strains (Fig. 7C).

To probe for a role for the sidechain in opsonization, parasites were incubated with diluted sera from chronically infected mice before being presented to murine BMDMs and allowed to either invade or be phagocytosed. Phagocytosis was scored microscopically as parasites co-localized with LAMP-1, which marks the phagolysosome, whereas invasion was differentiated as the formation of parasitophorous vacuoles marked by GRA7+. Although preincubation with chronically infected sera increased phagocytosis relative to naïve or no sera, no significant differences were detected between parental and PIGJ mutant CEP (Fig. 7D) or RH strains (Fig. S11C). To assay for neutralization by chronic serum of invasion of non-phagocytic cells, serum-coated parasites were incubated with MEFs and assessed for invasion relative to no serum coating by flow cytometry. No differences in antibody neutralization were observed between parental and PIGJ mutant CEP (Fig. 7E) or RH strains (Fig. S11D). Therefore, effector functions of immune and naïve sera are, within the sensitivity of our assays, unaffected in GPI sidechain null *T. gondii* parasites.

### The terminal glucose is required for immune serum IgM recognition of GIPL

**Fig 8:**
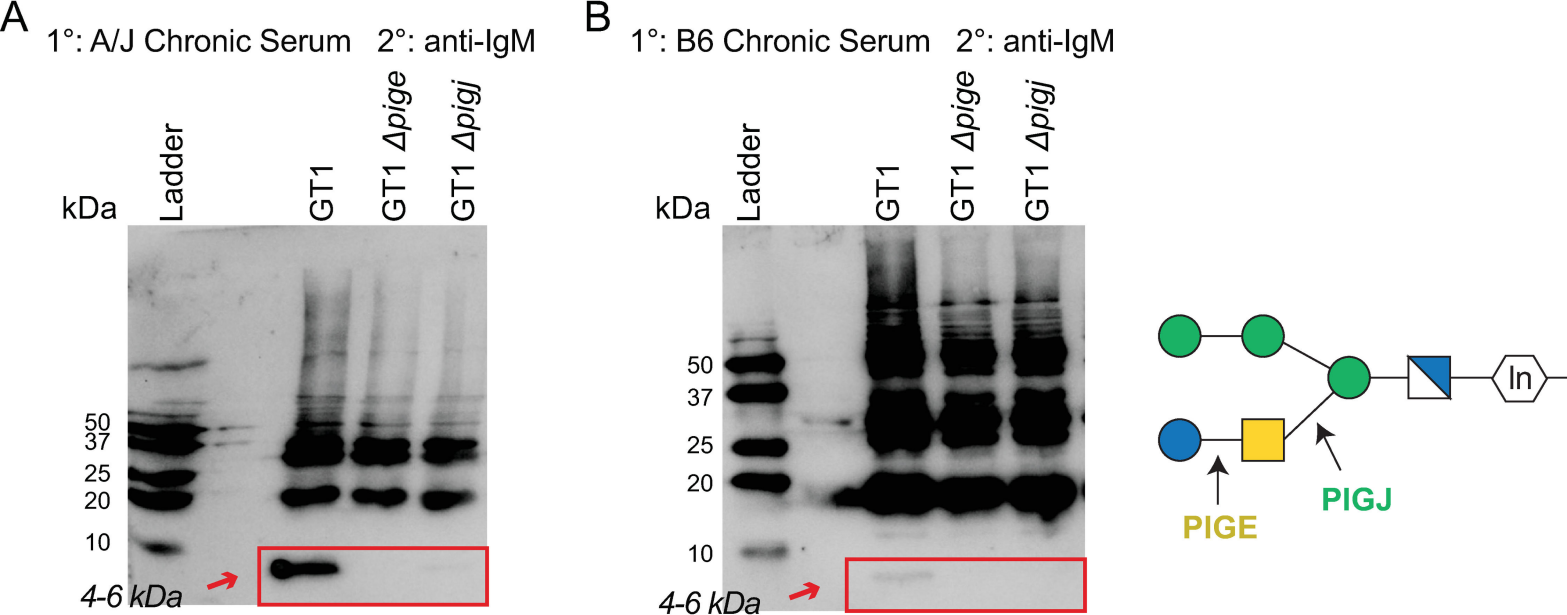
Anti-parasite immune serum IgM recognition of GIPLs requires the terminal sidechain glucose. The indicated whole parasite lysates were Western blotted and probed with serum from CEP chronically infected (**A**) A/J or (**B**) C57BL/6J (B6) mice. Anti-mouse IgM-HRP was used to detect IgM reactivity to the displayed parasite lysate antigens. Red boxes and arrows indicate the region where the low molecular weight 4–6 kDa GIPL antigen migrates. Schematic represents the *T. gondii* GPI with transferase activities of PIGJ and PIGE.

Prior evidence indicates that anti-GPI serum from humans latently infected with *T. gondii* is focused on the terminal Glc residue of the sidechain, based on probing of a synthetic phosphoglycan microarray (25). This preference was re-examined for murine sera based on the recognition of mutant GIPLs by Western blotting. Strong reactivity of serum from chronically infected A/J mice toward the GT1 GIPL band at 4–5 kDa was observed when assessed with anti-mouse IgM (Fig. 8A). IgM reactivity to GIPL was, however, absent in Δ*pige* and Δ*pigj* strains indicating the full sidechain is required for recognition, as previously inferred (21, 25). Similar findings were observed with C57BL/6J serum, though GIPL reaction intensity was weaker (Fig. 8B). Consistent with the flow cytometry data, antibody reactivity to parasite antigens 18 kDa and larger did not change between the wild-type and knockout parasites. Given the drop in serum IgM reactivity to both virulent (Δ*pigj*) and avirulent (Δ*pige*) strains, we reason this phenotype is unrelated to primary and secondary infection susceptibility of mice to GPI mutants.

### Non-significant macrophage tropism but enhanced CD36 binding of PIGJ mutants

**Fig 9:**
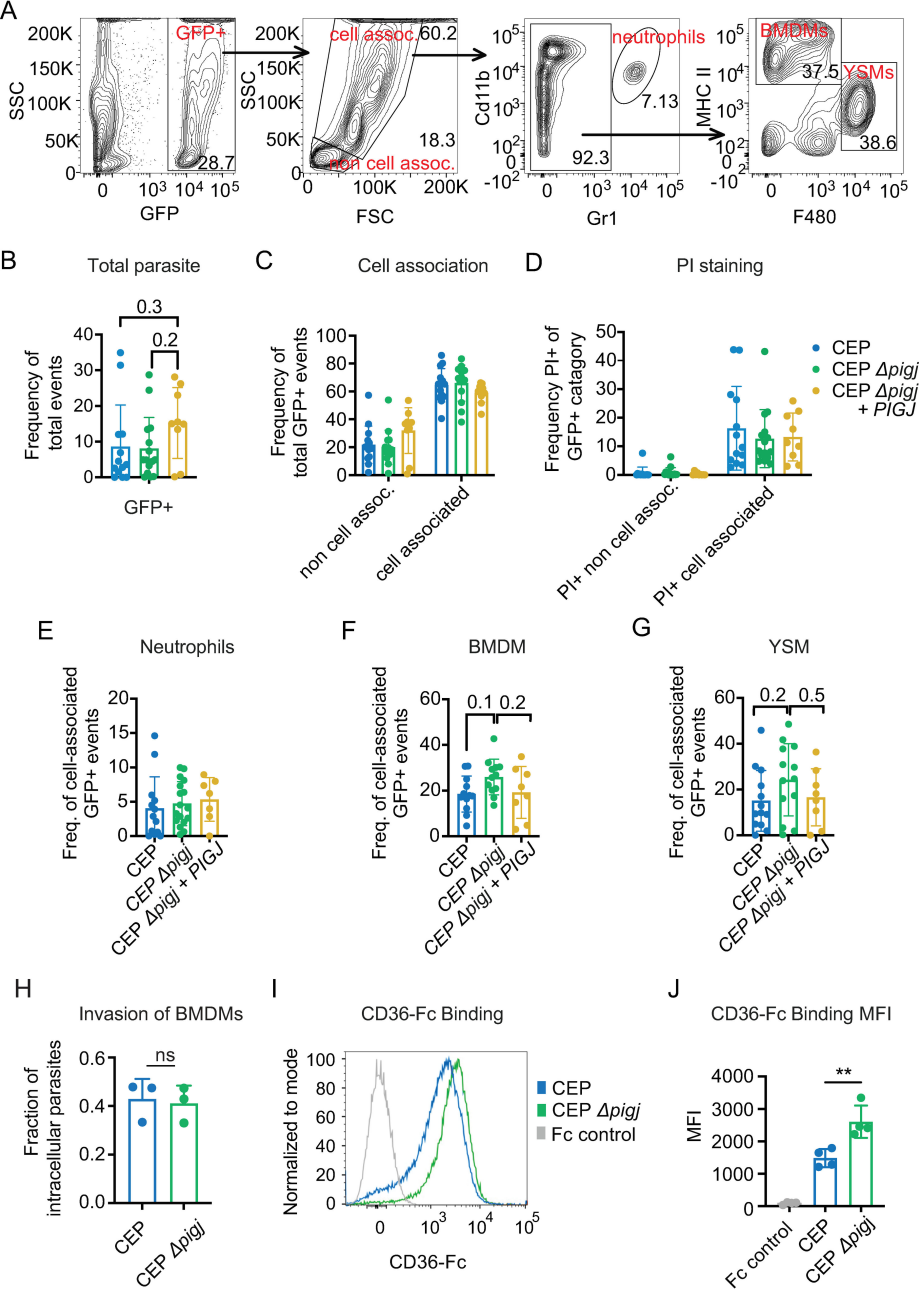
Enhanced CD36 binding and slight macrophage tropism 3 hours after infection. Mice (C57BL/6J) were injected i.p. with 10^6^ parasites of CEP, CEP Δ*pigj,* or CEP *Δpigj + PIGJ* and PECs were analyzed 3 hours later by flow cytometry. (**A**) Gating strategy for panels B–G. (**B**) Total GFP+ signal in the peritoneal exudate; GFP frequency of total events. (**C**) GFP+ events were separated by size using FSC and SSC parameters to determine extracellular or “non-cell-associated” and intracellular or “cell-associated” GFP+ parasites. (**D**) Frequency of PI+ (non-viable) cells of the indicated GFP+ category. (**E**) Frequency of cell-associated GFP+ events that were GR-1^hi^ Cd11b + neutrophils. (**F**) Frequency of cell-associated GFP+ events that were MHCII^hi^ F4/80^lo^ “BMDM,” or (**G**) MHCII^lo^ F4/80^hi^ “yolk sack-derived macrophages” (YSM). Plotted are seven total experiments; each dot represents a single mouse (mice; *n* = 12 CEP, *n* = 12 CEP Δ*pigj*, *n* = 8 CEP Δ*pigj + PIGJ*). Statistics performed were one-way ANOVAs with multiple comparisons and a Tukey correction; none were significant, and the values were indicated. (**H**) *In vitro* differentiated BMDMs were plated on coverslips overnight and infected with 100 and 300 GFP+ parasites per well, and parasites were allowed 20 minutes to invade cells before being fixed and stained for fluorescence microscopy. No permeabilization was used, and SAG1 staining was performed. Intracellular parasites were not stained with SAG1 but were GFP+, while extracellular parasites are SAG1+GFP+ . The fraction of intracellular parasites is plotted, dots represent independent experiments; *n* = 3 experiments. (**I**) Parasites were incubated for 1 hour with recombinant CD36-Fc and CD36 binding was measured with anti-human-IgG-Daylight-550 via flow cytometry. Representative histogram and (**J**) average + SD MFI of CD36 binding from four experiments is plotted with each experiment represented as a dot, and an unpaired t-test is shown, **P < 0.01. For I and J, also plotted are IgG Fc staining controls of the CEP strain (Fc control).

To test whether cellular tropism of GFP+ expressing parasites may explain PIGJ-mediated differences in host susceptibility to infection, mice were infected with 10^6^ parasites and 3 hours later peritoneal exudate cells (PECs) were analyzed by flow cytometry as previously described (65). Invasion of PECs was estimated by analyzing the forward scatter (FSC) and side scatter (SSC) parameters of GFP+ events to differentiate by size and granularity (Fig. 9A). The three strains, CEP, CEP Δ*pigj*, and CEP Δ*pigj + PIGJ_3×HA_*, produced similar GFP signal (Fig. 9B), and no differences in cell-associated (FSC^int-hi^ SSC^int-hi^) vs non-cell-associated (FSC^lo^ SSC^lo^) GFP+ parasites were observed (Fig. 9C), the latter gate likely representing small extracellular parasites. Cell death, as inferred by PI staining, was also similar between parasites, suggesting similar death kinetics of parasite-infected peritoneal cells (Fig. 9D).

The cell-associated GFP+ events were further analyzed for markers to identify infected cell types to assess cell-type tropism. Gr-1^hi^ Cd11b + neutrophils had no significant differences in infection frequencies of the three parasite strains (Fig. 9E). Similarly, there was no significant differences in the infection frequency of bone marrow-derived macrophages (MHCII^hi^ F4/80^lo^ “BMDM” cells) (Fig. 9F), or for MHCII^hi^, F4/80^hi^ tissue-resident or yolk-sack macrophages (“YSM”) (Fig. 9G). Nevertheless, though lacking statistical support, increased frequencies of parasitized macrophages trended for CEP Δ*pigj* parasites suggesting potential tropism toward BMDM or YSM cells. This trend appeared unrelated to invasion, as similar invasion frequencies of *in vitro* differentiated BMDMs were observed between the parental and Δ*pigj* strains (Fig. 9H).

*T. gondii* tropism for macrophages is mediated in part by CD36 (65), a scavenger receptor expressed in many tissues and immune cells that plays a role in fatty acid uptake, angiogenesis, and phagocytosis (66). Therefore, parasites were incubated with recombinant CD36, and binding was measured via flow cytometry. CD36 binding to CEP Δ*pigj* compared to wild-type CEP strains was significantly increased (Fig. 9I and J). Whether this difference is associated with increased virulence of CEP Δ*pigj* parasites deserves further study.

### Virulence of Δ*pigj* strains is galectin-3 and sex dependent, but not TLR-2 nor -4 dependent

**Fig 10:**
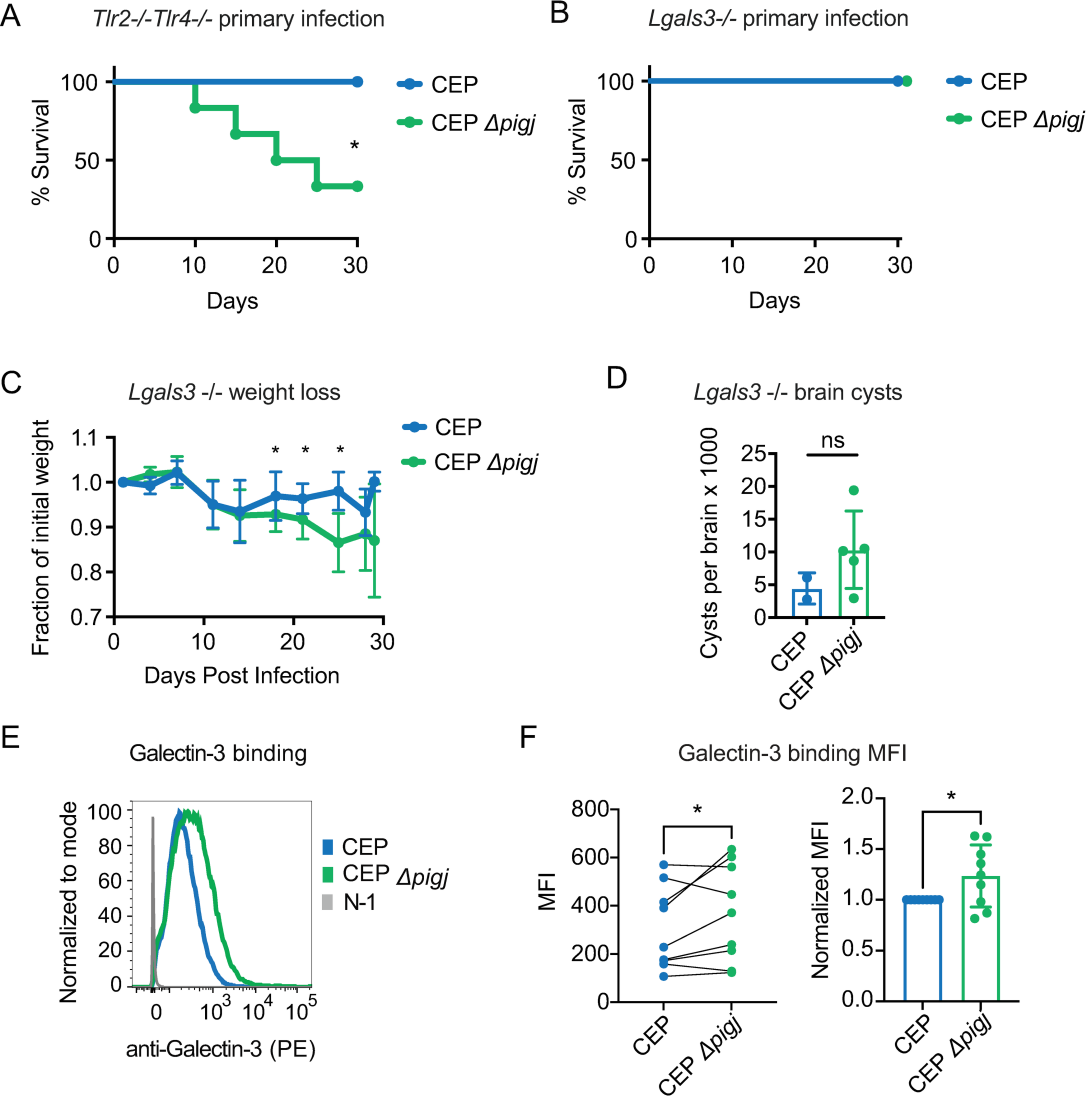
CEP Δ*pigj* virulence is galectin-3 dependent. (**A**) *Tlr2/4*−/− mice were given primary infections (i.p.) of 10^4^ parasites of CEP or CEP Δ*pigj* and monitored for survival for 30 days. Plotted are cumulative results from two experiments (mice; *n* = 5 CEP, *n* = 6 CEP Δ*pigj*); log-rank (Mantel-Cox), *P < 0.05. (**B**) *Lgals3*−/− mice were given primary infections of 10^4^ parasites of CEP or CEP Δ*pigj* and monitored for survival for 30 days. Plotted are the cumulative results from four experiments (mice; *n* = 15 CEP, *n* = 23 CEP Δ*pigj*). (**C**) Plotted is the average ±SD fraction of initial weight for each cohort analyzed in B. Significance was determined by one-way ANOVA test, *P < 0.05. (**D**) Brain cysts were quantified from surviving mice in panel B. Shown are two experiments (mice; *n* = 2 CEP, *n* = 5 CEP Δ*pigj*); unpaired t-tests were performed, ns = non-significant. (**E**) Whole parasites were incubated with 10 ug/mL of recombinant mouse galectin-3 and galectin-3 binding was detected with anti-Gal-3-PE by flow cytometry. Representative histogram of galectin-3 binding; N-1 control is the staining in the absence of galectin-3. (**F**) MFIs of galectin-3 parasite binding from nine experiments are plotted and each experiment is represented by a dot. MFI values are connected by experiment. Normalized MFIs values (CEP = 1) are also plotted. Statistical analysis was performed with an unpaired, one-tailed t-test; *P < 0.05.

Since TLR2 and TLR4 (17) and galectin-3 recognize the *T. gondii* GPI (18), we hypothesized that mice lacking these TLRs or galectin-3 would render them refractory to disease modulation by the GPI sidechain. First, *Tlr2/Tlr4*−/− double knockout mice were used to study the survival of the CEP Δ*pigj* strain. Like wild-type animals (Fig. 4A), the double knockout mice succumbed to CEP Δ*pigj* while surviving CEP infections (Fig. 10A), suggesting that differences in the GPI sidechain do not impact host-parasite interactions mediated by these pattern recognition receptors. By contrast, mice lacking the gene for galectin-3, *Lgals3*−/−, survived both CEP and CEP Δ*pigj* infections (Fig. 10B). The CEP *Δpigj* infected mice did not have significant differences in brain cyst burdens (Fig. 10D), though they did experience significant weight loss later in the infection (Fig. 10C). This indicates that the Δ*pigj* virulence observed in wild-type mice is dependent on galectin-3. Furthermore, an increase in recombinant galectin-3 binding to CEP Δ*pigj* parasites was observed via flow cytometry (Fig. 10E and F). Thus, the absence of the GPI sidechain is a candidate for explaining increased galectin-3 binding to parasites and increased parasite virulence.

Notably, the virulence of CEP Δ*pigj* parasites toward female C57BL/6J mice was not observed in male counterparts, based on survival to infection (Fig. S12A). Male mice were also protected from secondary infections with RH Δ*pigj* mutants (Fig. S12C). These mice sustained their weight similarly to the wild-type-infected mice (Fig. S12B and D). There are sex differences in a variety of immune responses (67), including susceptibility to acute *T. gondii* infections, and brain cyst burdens (68). The possibility that the GPI sidechain is mechanistically connected to sex-dependent virulence requires further investigation.

## DISCUSSION

This study aimed to define the role of the GPI sidechain in parasite virulence by identifying and mutating its GPI sidechain-modifying enzymes. In our attempts to answer these questions, we made several critical observations. First, we identified the two glycosyltransferases responsible for GPI sidechain modification in *T. gondii*, *Tg_207750*, named PIGJ, which adds the GalNAc to the mannose of the core backbone, and *Tg_266320*, named PIGE, which adds the glucose to the GalNAc sidechain. We confirmed their activity using mass spectrometry analysis of the GPI in the knockout parasites, which clarified ambiguities using monoclonal antibodies which apparently have some cross-reactivity to unknown epitopes (Fig. S4). In addition to the characterization of these GTs, we explored their role in parasite virulence in both primary and secondary infections which revealed that PIGJ mutants are virulent in both settings. This study is a first analysis for the role of the GPI sidechain for any microbe in pathogenesis.

Removal of the core GPI synthesizing enzymes is lethal in mammals and in eukaryotic microbes. However, it remained an outstanding question of whether complete removal of the GPI sidechain would have fitness bearing impacts in parasites. Deletion of the proteins identified here does not have deleterious impacts on the lytic cycle, and the fitness scores for PIGJ and PIGE align with this conclusion (Fig. 1B). In performing reciprocal blasts using *T. gondii* PIGJ and PIGE, we find evidence that homologous enzymes exist in coccidian species, but not in more distantly related apicomplexan species such as *Plasmodium* and *Cryptosporidium* (not shown). Coccidia parasites are orally acquired parasites of warm-blooded animals that have an intra-epithelial stage in their development, but many form tissue cysts, which require parasite dissemination following infection. Whether all coccidia parasites express similar Glc-GalNAc-sidechains is unknown but would be expected from the identification of PIGJ and PIGE homologs across this subclass of parasites. Intriguingly, PIGJ evolved independently of the mammalian PGAP4, which catalyzes the same reaction in the animal Golgi (33, 34). PGAP4 is a CAZy GT109 family member that bears no recognizable homology with CAZy GT17 sequences and has a distinct architecture that includes a transmembrane hairpin inserted within the canonical catalytic domain. This outstanding example of convergent evolution tempts speculation that the parasite uses this similarity to mimic a biological process mediated by this glycoform in the host. Its inferred type 2 membrane protein classification indicates that the catalytic domain functions in the lumen of the secretory pathway. The contrary evidence that sidechain glycoforms of GPIs can be detected on the cytoplasmic surface of secretory organelles (59) might alternatively be explained by the presence of scramblases in the rER (69). Given that type 2 membrane GTs and UDP-GalNAc transporters are typically found in the Golgi invites speculation that, like PGAP4, PIGJ may function in the Golgi downstream of assembly of the glycan core and after further processing and transfer to proteins within the ER. However, fluorescence microscopy suggests PIGJ_3×HA_ is enriched in the rER (Fig. 5). PIGJ is clearly a late step in GPI processing considering its assembly as a GIPL and an anchor in *PIGJ* deletion strains, but whether that occurs in the rER or the Golgi in the parasite is considered unresolved because of caveats associated with analysis of an overexpressed and epitope-tagged protein.

The most similar GT found for PIGE is lactosylceramide 4-alpha-galactosyltransferase (a.k.a. globotriaosylceramide or Gb3 synthase), which attaches αGal to the 4-position of a β-linked Gal acceptor across many eukaryotes (70). Thus, the linkage formed is the same, but the donor and acceptor sugars are distinct, which is an evolutionary variation common among CAZy GT32 GTs. GT32 GTs utilize sugar nucleotide rather than Dol-P-sugar donors, as previously documented for the enzyme that assembles the Glcα1,4-linkage in the *Toxoplasma* GPI (50). Though PIGE and Gb3 synthase are both type 2 membrane proteins that modify glycolipids, PIGE is distinctive with its ~385 aa long stem-like region and a ~160 aa poorly conserved, likely disordered cytoplasmic region, whereas Gb3 synthase is only 360 aa overall with a negligible cytoplasmic region. PIGE also encodes a DxD-like EDD motif in its GT domain as found in PGAP4 (33) (Fig. 1A), though its significance remains unresolved.

In our search for PIGJ and PIGE functions, their genes were edited to prevent the expression of their proteins in both type I (GT1, RH) and type III (CEP) strains of *T. gondii*. Parasites lacking PIGJ, but not those lacking PIGE, exhibit increased virulence in primary and secondary infections, demonstrating the GPI sidechain modulates parasite virulence by preventing lethality in its host. To elucidate the mechanism for increased host susceptibility to the PIGJ mutant we characterized a variety of immune parameters and parasite burden following infection. Our findings indicate early parasite burden in the peritoneal cavity, liver, and lung are similar, yet the PIGJ mutants can evade clearance and establish higher cyst burdens in the brain. Cytokine responses, antibody reactivity, opsonization, neutralization, and parasite surface antigen expression were all similar between strains that express a GPI sidechain and those that do not. We speculate that a breakdown of infectious tolerance occurs early during infection when the parasite lacks a GPI sidechain. Whether enhanced tissue damage results following infection with GPI sidechain null parasites is unknown.

To further explore where the pathogenesis might take place, CD36 binding was found to be increased with CEP Δ*pigj*, which might be associated with a trend toward increased albeit insignificant cell tropism for macrophages in the peritoneal cavity 3 hours after infection. We also found that *Lgals3*−/− mice were protected against primary infections with CEP Δ*pigj*, indicating a fundamental role of galectin-3 in the susceptibility of wild-type mice to CEP Δ*pigj* infections. Galectin-3 is one of the few known host factors that bind *T. gondii* GIPL (18), and recombinant galectin-3 preferentially bound CEP Δ*pigj* strains. Galectin-3 was previously shown to be required for macrophage TNFα production in response to *T. gondii*, and it was proposed that galectin-3 might act as a co-receptor that presents the GPI to TLR2 (18). Our data of PIGJ infections in double knockout *Tlr2/4*−*/*− mice indicated that TLR2/4 signaling was not involved in the virulence differences. There are likely other cellular functions mediated by galectin-3 binding to GIPL that do not involve TLR2/4; however, the exact mechanisms are unknown. Previous reports have shown that following *T. gondii* infection, galectin-3 is upregulated in peripheral tissues during acute infection, and *Lgals3*−/− mice were susceptible to intraperitoneal infections with type II strains (20). Interestingly, *Lgals3*−/− mice had higher Th1 responses with increased IL-12p40 and IFNγ detected in the sera, as well as from cultured splenocytes after infection (20). It is possible that the enhanced Th1 response underpins the resistance of *Lgals3*−/− mice to type III strains but renders them susceptible to more virulent type II strains. Regardless, further mechanistic insight is required to draw correlations between galectin-3 and the GPI sidechain and to place this interaction as central to the disease outcome of PIGJ mutants.

Of further note on the CD36 pathway, it is possible that mice infected with Δ*pigj* strains are more able to phagocytose parasites via CD36, allowing for enhanced infection and escape via the phagosome cellular-invasion route reported for less virulent parasite strains (71). *Cd36*−/− susceptibility to *T. gondii* infections was suggested to correlate with a breakdown in tissue homeostasis following infection (65). This was revealed by monitoring serum indicators of tissue stress including GDF-15 and FGF21. GDF-15 induces anorexia by binding to the GFRAL receptor expressed by neurons of the hindbrain (72), while GFG21 is a hormone that controls energy homeostasis and adiposity (73) and is often induced in multiple tissues by nutrient starvation and endoplasmic reticulum stress (74). Given that the PIGJ mutants appear normal with respect to parasite burden during the first week of infection and the disease outcome is sex linked, we suspect the GPI sidechain promotes tissue homeostasis by potentially controlling mechanisms of tolerance. This tolerance may be mediated via altered CD36 or galectin-3 interactions with the GPI sidechain and/or other host pathways yet to be identified.

In our initial approach to confirm the loss of the Glc and GalNAc sidechain additions, we utilized glycoform-specific antibodies T3 3F12 and T5 4E10. While complete removal of the sidechain in the Δ*pigj* strains was clearly observed through Western blotting, the removal of the glucose addition was not. The T5 4E10 antibody, though it was initially described to be specific for the GalNAc + Glc glycoform (21), has considerable cross-reactivity to the GalNAc-only glycoform, as seen in previous studies (21, 33). The T3 3F12 antibody clone is an IgG3 isotype, and the T5 4E10 clone is an IgM isotype. It is interesting to note that B-1 cells preferentially produce antibodies of IgM and IgG3 isotypes. B-1 cells are responsible for natural self-reactive IgM antibodies and are known to contribute to T-independent responses, many of which are against non-protein antigens (75). We originally considered the GPI as a non-protein antigen after we found that B-1 cells are important for immunity to secondary infections of *T. gondii* (54). With respect to the IgM response, we did note that IgM recognition of GIPL was highly sensitive to the terminal glucose (Fig. 8), as previously observed using chemically synthesized GPI and serum from latently infected humans (25). Whether the antibody response to GIPL is from B-1 cells is unknown. Regardless, the lack of antibody reactivity to GIPLs derived from PIGE and PIGJ mutants cannot account for why these strains differ in primary and secondary infection virulence.

The analysis of the type I RH strain presents additional insights into the significance of the sidechain and its biosynthesis. Though previous reports indicated the detection of RH GIPL and its sidechain (21), our recent report using mass spectrometry of RH GPI-AP revealed only the linear mannose core of GPI-anchors with no sidechain (38) and confirmed here in a separate RH strain. A potential cause for this could be the laboratory adaptation of this strain. This strain was initially passaged in mice and then in tissue culture for over 60 years before being frozen down. Due to this, the RH strain has become laboratory adapted, and acquired some unique characteristics (48). For instance, RH has lost its ability to form orally infectious tissue cysts, grows much more rapidly than other strains, and has increased extracellular viability (76, 77). We have discovered that the type I RH strain has lost its ability to evade immunological memory responses that are generated following vaccination or natural infection, unlike other type I strains like GT1 (46, 54). It is possible that for whatever reason, RH has lost its expression of the GPI sidechain through this laboratory evolution. However, it was an initial report that the gene *Tg_207750* was almost six times more highly expressed in RH compared to GT1 that led us to investigate it as a GPI sidechain GT candidate (48). A second possible explanation is an identified non-synonymous SNP that generates an L620R substitution uniquely in this strain relative to all other available sequences (Fig. 1A) (not shown). This position is conserved in a hydrophobic core of this region of the enzyme that is involved in coordinating a PO_4_ group of the UDP-GalNAc substrate. Whether this acquired mutation in RH inactivates PIGJ, which, in turn, leads this strain to compensate by overexpressing the gene is unknown. Interestingly, full deletion of the gene increased virulence in secondary infections (Fig. 4C), suggesting an additional role for PIGJ that might be mediated by other regions of the protein. It is possible that PIGJ, like most other enzymes in the GPI processing pathway (78), belongs to a multiprotein complex that depends in part on the presence of the PIGJ protein, and that its absence disturbs some other aspects of GPI anchor assembly such as selectivity or efficiency.

In conclusion, this study identified and characterized both GPI sidechain-modifying GTs of *T. gondii*. In addition, for the first time in any microbe, the effects of the complete loss of the sidechain were studied for its role in pathogenicity. The results presented here indicate a fundamental role of the presence of the sidechain in host survival and that when the sidechain of the GPI is lost, so too is host resistance to *T. gondii*. Although most of the major surface antigens of *T. gondii* are GPI-anchored SAGs and the GPI is known to be targeted by antibody responses in a variety of protozoan infections (23, 30), our findings here would suggest that for *T. gondii* antigens, the sidechain presence does not impact antibody reactivity to GPI-anchored proteins. However, whatever other humoral functions are at play regarding host recognition of GIPL, they are disrupted when the sidechain is lost. We suggest both a fundamental, and yet diverse role for the GPI sidechain in microbial pathogenesis. Based on our data, we propose a model in which enhanced CD36 and galectin-3 binding of PIGJ mutants lead to pathology, perhaps through differences in cellular tropism and unknown inflammatory mediators yet to be defined (Fig. S13).

## SUPPLEMENTAL MATERIAL

Supplemental Figures mbio.00527-24-s0001.docx Figures S1-S13.

Table S1 mbio.00527-24-s0002.xlsx Parasite strains, oligos, and plasmids.

ASM does not own the copyrights to Supplemental Material that may be linked to, or accessed through, an article. The authors have granted ASM a non-exclusive, world-wide license to publish the Supplemental Material files. Please contact the corresponding author directly for reuse.
