# Supplementary material for: The GPI sidechain of Toxoplasma gondii inhibits parasite pathogenesis
Source: mBio. 2024 Sep 20;15(10):e00527-24. doi: 10.1128/mbio.00527-24 (PMC11481522; doi:10.1128/mbio.00527-24)
Supplement: Supplemental Figures — Figures S1-S13. [file mbio.00527-24-s0001.docx]

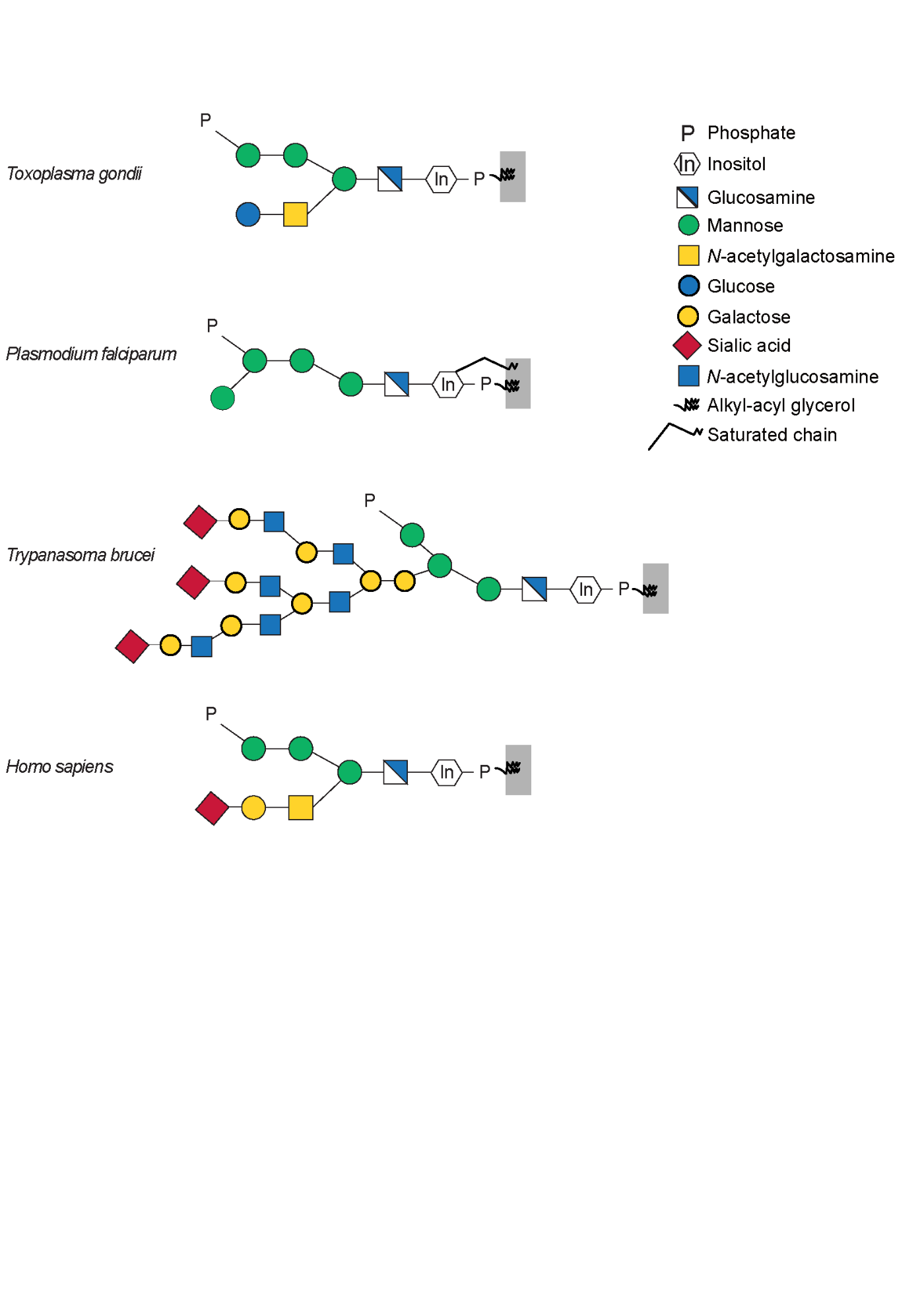


**Figure S1: GPI sidechains differ between eukaryotic species.**

Schematic of the diversity of GPI sidechains between species (*T. gondii*, *P. falciparum*, *T. brucei* procyclic stage, *H. sapiens*). Note the conserved mannose backbone with species variability of sidechain modifications. Not all GPI glycoforms for each species are represented.


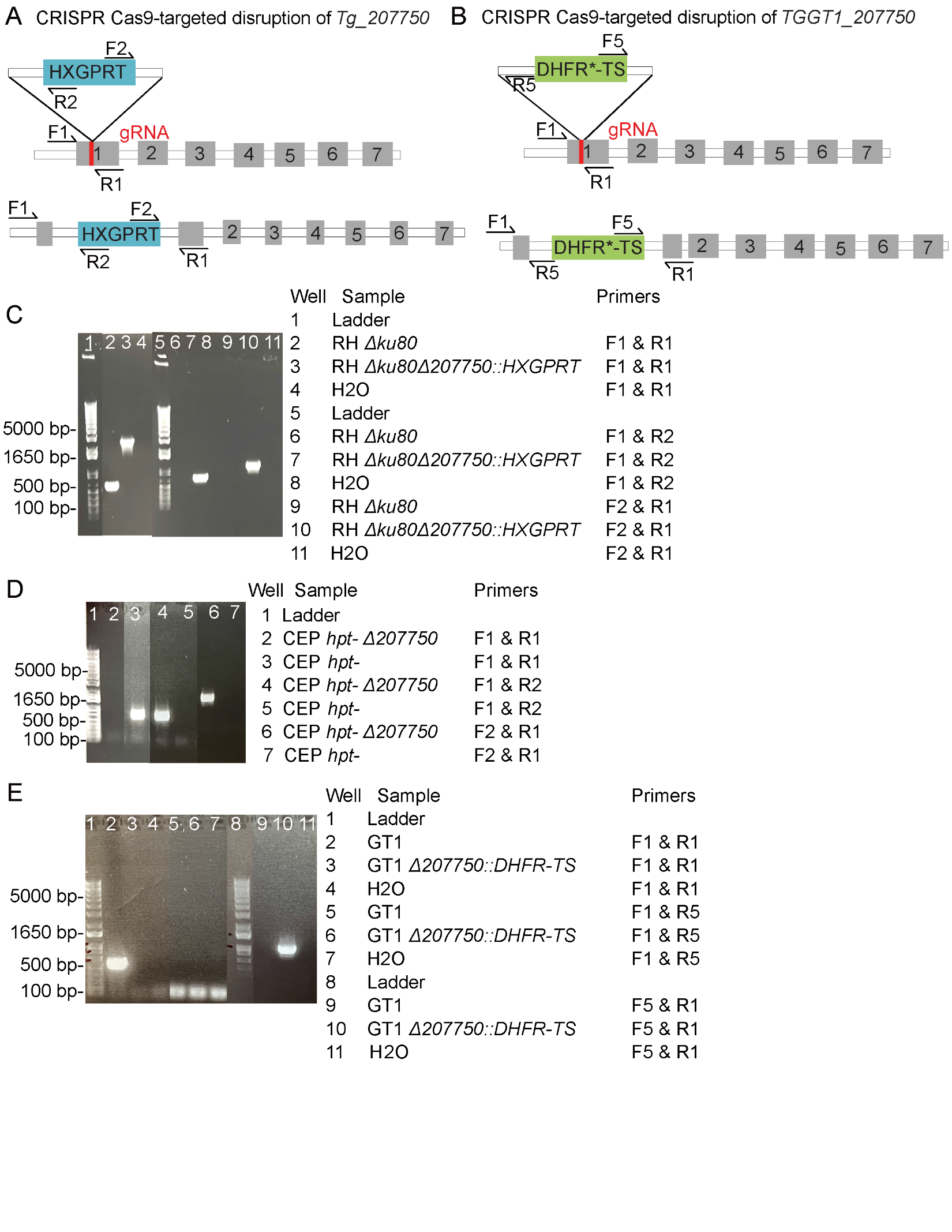


**Figure S2: Approach and PCR confirmation of *Tg_207750* disruption.**

Schematic of the CRISPR Cas9 targeted disruption of the *Tg_207750* (*PIGJ*) locus and insertion of the selectable marker *HXGPRT* in A, or *DHFR-TS** in B. C-E) PCR confirmation of disruption of the targeted locus and insertion of the selectable marker within the cut site using the primers indicated in panels A or B.


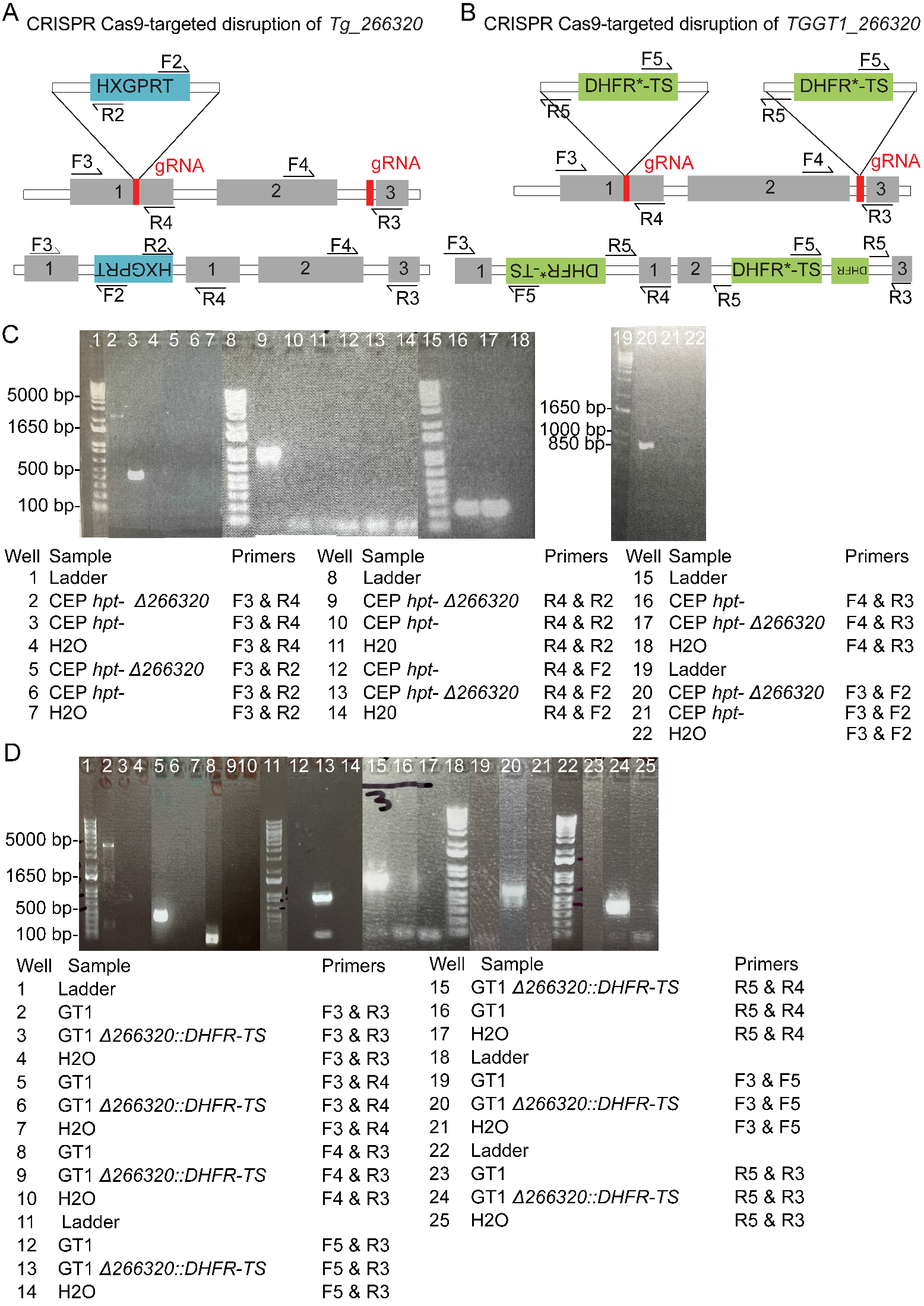


**Figure S3: Approach and PCR confirmation of *Tg_266320* disruption.**

Schematic of the CRISPR Cas9 targeted disruption of the T*GGT1_266320* (*PIGE*) locus and insertion of the selectable marker *HXGPRT* in A, or *DHFR-TS** in B. C-D) PCR confirmation of disruption of the target locus and insertion of the selectable marker within the cut site using primers in A or B. In the case of CEP *Δ266320*, the *HXGPRT* selectible marker inserted into the Cas9 cut site in exon one in reverse orientation, but not the second site in exon 3. In the case of GT1 *Δ266320*, the *DHFR-TS** selectible marker inseted in reverse orietnation in the Cas9 cut site in exon 1, while the cut site in exon 3 was repaired with a concatemerized *DHFR-TS** as prposed based upon the diagnostic PCR.


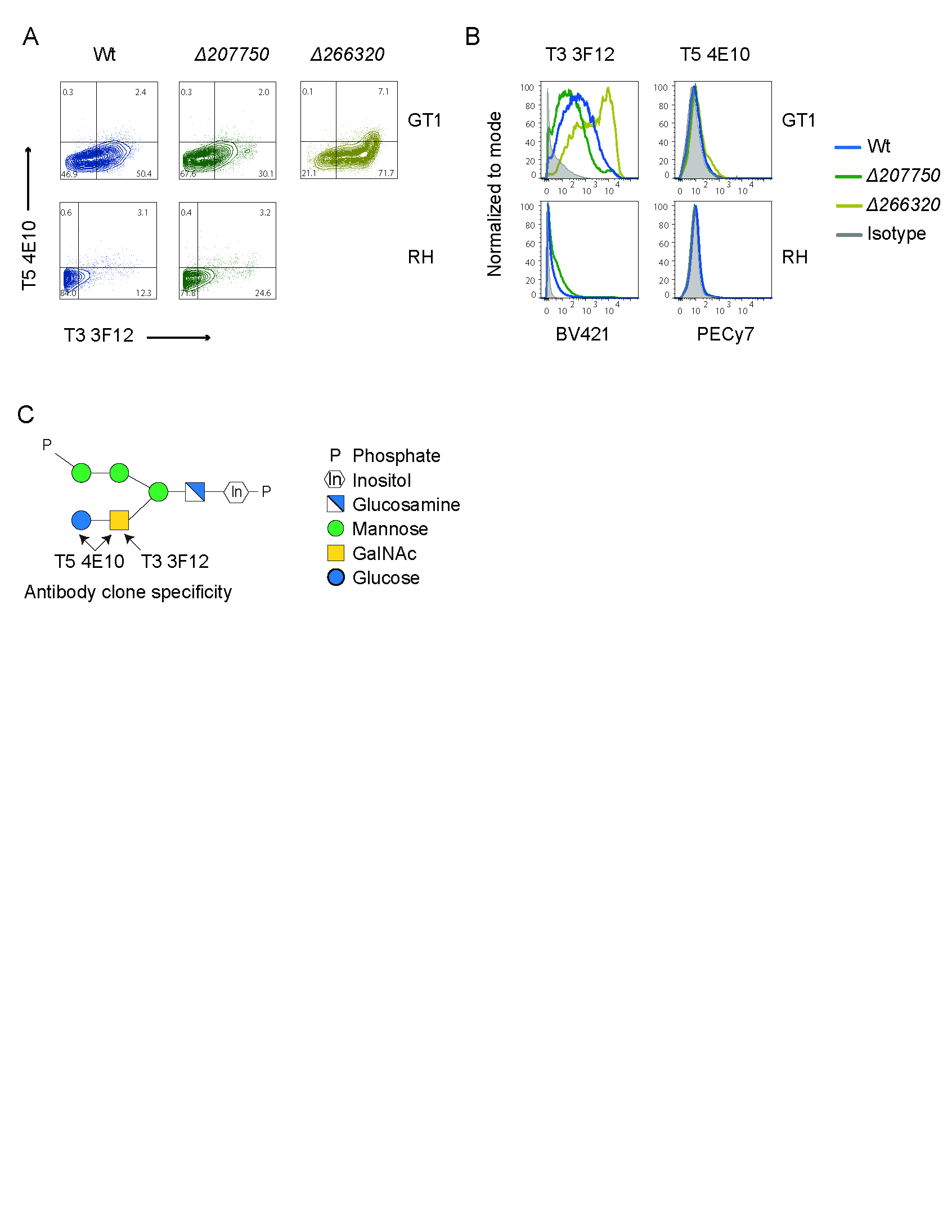


**Figure S4: Decreased T3 3F12 recognition of the GT1** ***Δpigj* and RH strains by flow cytometry.**

A) Fixed parasites of the indicated type I GT1 and RH parasite strains were stained with GIPL glycoform-specific antibodies T3 3F12 (mouse IgG3) and T5 4E10 (mouse IgM). Secondary fluorescent anti-isotype antibodies were used to detect T3 3F12 and T5 4E10 binding. SAG3 positive parasites were analyzed to distinguish parasites from debris. Data is representative of 4-9 experiments. B) Representative histograms from the flow cytometry analysis comparing the parental with the mutant strains of the data shown in A. In addition, a isotype staining control is plotted for comparison (grey histogram), which were used to set the quadrants in panel A. C) Schematic indicating the GIPL glycoform specificity of antibody clones T3 3F12 (binds the GalNAc glycoform) and T5 4E10 (binds both GalNAc +/- Glc glycoforms).


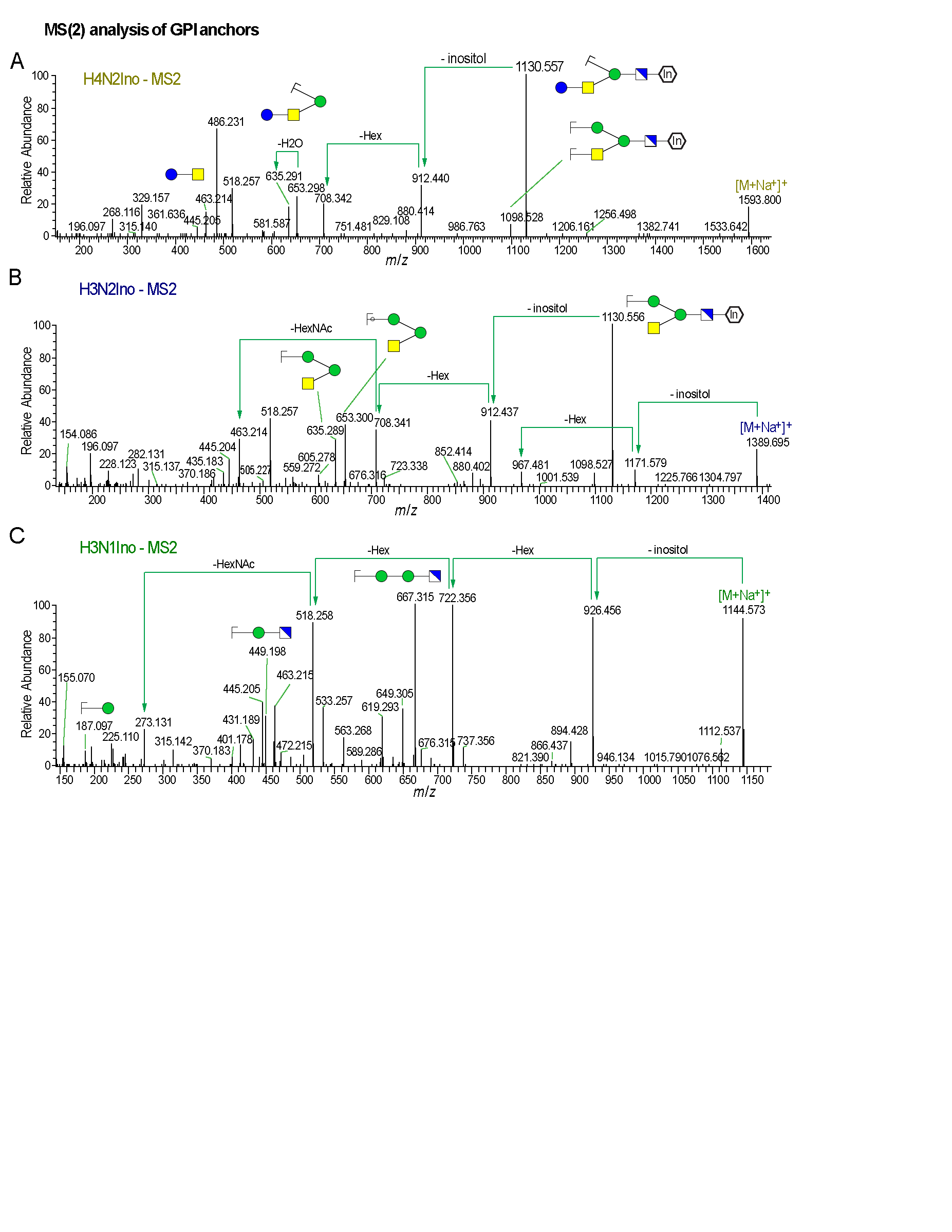


**Figure S5: Fragmentation analysis of GPI-anchor glycans.**

Isolated primary GPI ions were selected in MS(1) as described in Figure 3 and subjected to collision-based MS(2) fragmentation during the nLC-MS run. Residual parent ions are labeled at the high m/z end, and decomposition products at lower m/z values. Green arrows trace sequential fragmentation pathways. A) H4N2 glycan from strain GT1. B) H3N2 glycan from GT1 *Δpige*. C) H3N1 from GT1 *Δpigj*.


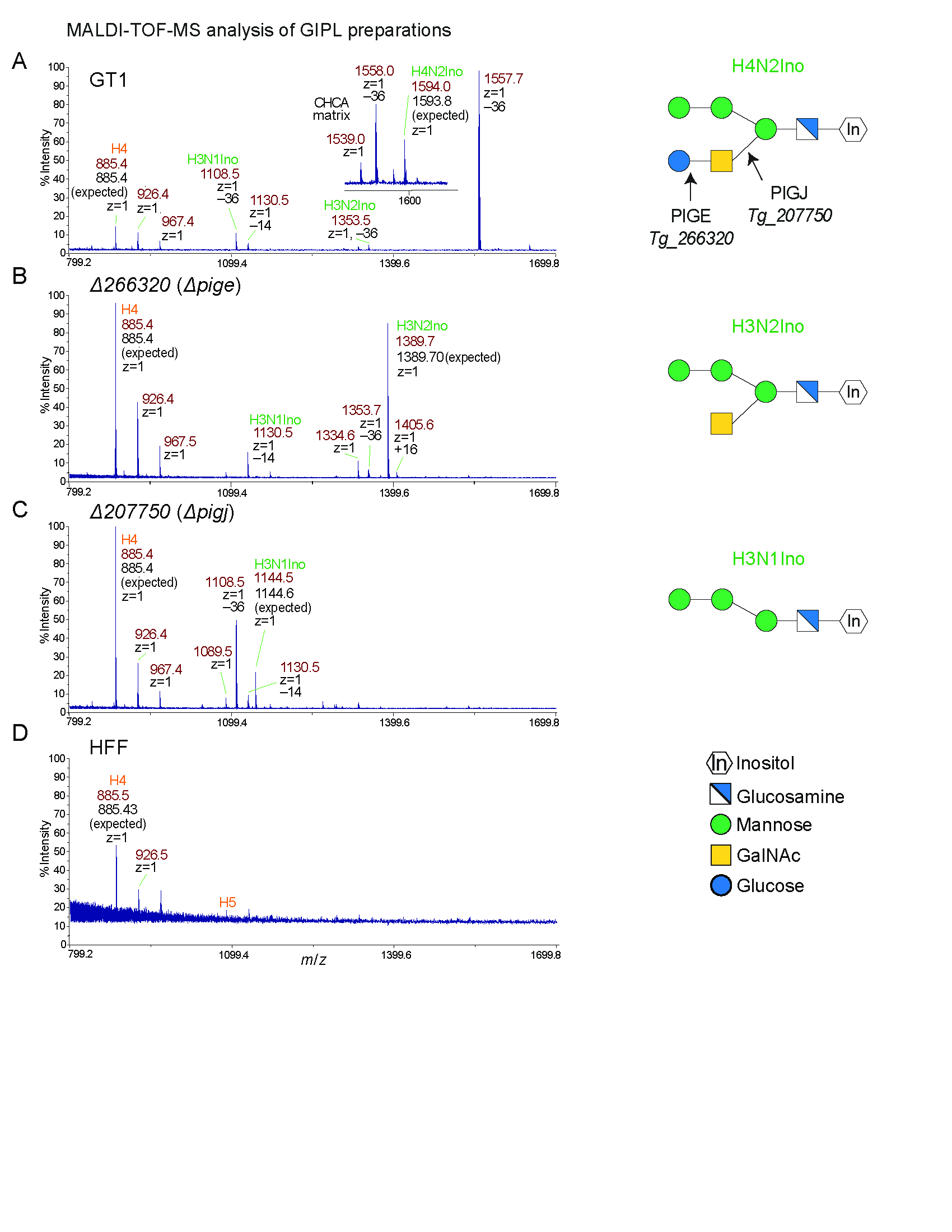


**Figure S6: MALDI-TOF-MS analysis of GIPL preparations is consistent with PIGE and PIGJ as being the GPI sidechain glycosyl transferases in *T. gondii*.**

Glycans isolated from GIPL samples from tachyzoite stage parasites were N-acetylated and permethylated, and mixed with DHB matrix and analyzed as in Figure 2. A) Parental GT1 strain. The inset shows a sample prepared in CHCA rather than DHB matrix. See text for the basis of assignment of ions that differ by a m/z defect of -36. B) *Δpige*. C) *Δpigj*. D) Host cells (HFFs). Schematics indicating the *T. gondii* GPI with confirmed GT assingments for PIGJ and PIGE, and corresponding hexose composition of the various glycans.


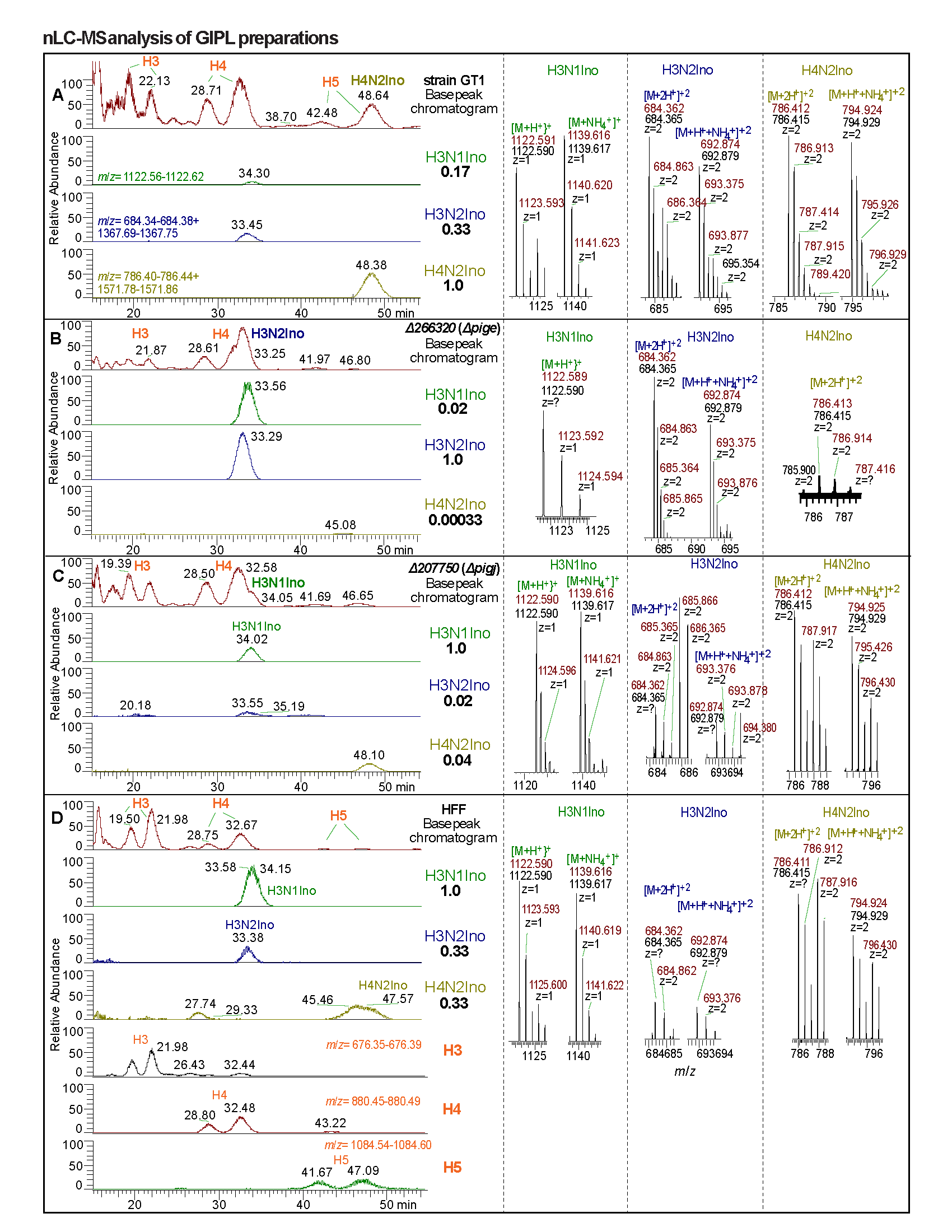


**Figure S7: nLC-MS analysis of GIPL preparations is consistent with PIGE and PIGJ as being the GPI sidechain glycosyl transferases in *T. gondii*.**

Isolated GIPL fractions described in Figure S6 were reanalyzed by nLC separation on a C18 column and hyphenated analysis in an Orbitrap mass spectrometer in positive ion mode. The left-hand column of panels shows base peak chromatograms (all ions m/z 500-2000) and extracted ion chromatograms (EIC) for each of the indicated targets (m/z ranges given in panels A and D). The ratio of ion intensities over the EIC m/z ranges shown for H3N1Ino, H3N2Ino, and H4N2Ino are shown relative to the most abundant ion. The most abundant ions are labeled in the base peak chromatogram. The right-hand column of panels shows representative mass spectra of the one or two most abundant ions eluting with the target and used for quantitating relative levels. Levels of hexosamers were not quantitated. Observed m/z values are in dark red, expected values are in black, and the charge state is as indicated. A) Parental GT1 strain. B) *Δpige*. C) *Δpigj*. D) Host cells (HFFs).


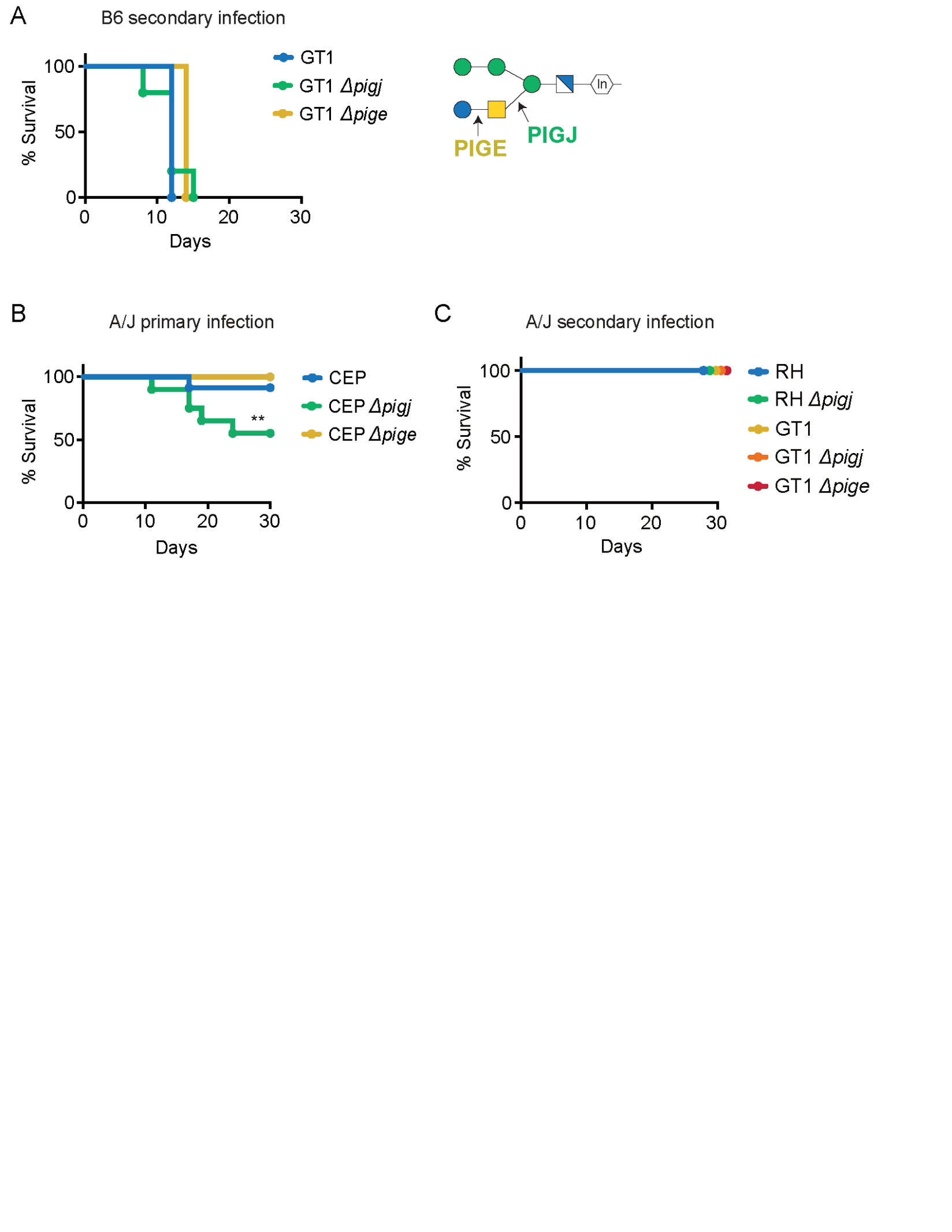
 **Figure S8: Primary and secondary infections with various PIGJ and PIGE mutants in C57BL/6J and A/J mice reveal a fundamental role for PIGJ in primary infections.**

A) C57BL/6J (B6) mice given a primary infection with the avirulent type III strain CEP, 35 days later were given a secondary infection with 5x10^5^ parasites of the type I strain GT1, GT1 *Δpigj* or GT1 *Δpige* strains and tracked for survival. Plotted is the result from one experiment (mice; n = 3 GT1, n=5 GT1 *Δpigj*, n=1 GT1 *Δpige*). Schematic indicating the *T. gondii* GPI with transferase activities of PIGJ and PIGE. B) A/J mice were given primary infections of 10^4^ parasites of either CEP, CEP *Δpigj* or CEP *Δpige* and monitored for survival for 30 days. Cumulative survival from 1-3 experiments is plotted (mice; n = 23 CEP, n = 20 CEP *Δpigj*, n = 6 CEP *Δpige*). C) After A/J mice were given a primary infection with the avirulent type III strain CEP, 35 days later were given a secondary infection with 5x10^4^ parasites of the indicated type I strains and tracked for survival. A single experiment was performed (mice; n = 5 RH, n = 3 RH *Δpigj*, n = 2 GT1, n = 5 GT1 *Δpigj*, n = 10 GT1 *Δpige*). For survival analysis, significance was determined by Log-rank (Mantel-Cox) test, ** p<0.01.


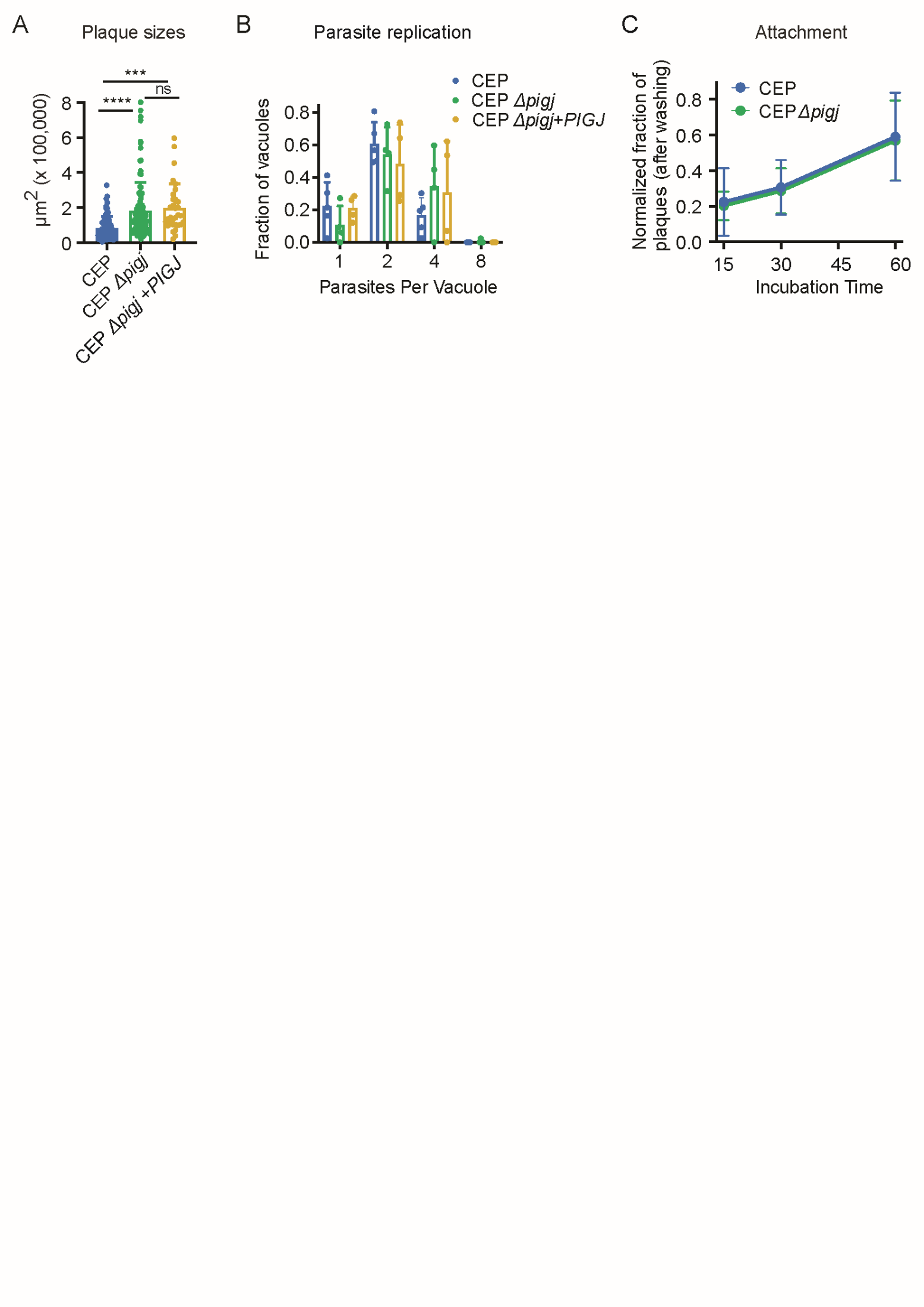


**Figure S9: PIGJ mutants have no apparent fitness defects *in vitro*.**

A) HFF cells in 24 well plates were infected with either CEP, CEP *Δpigj*, or CEP *Δpigj* + *PIGJ* and allowed to grow for 5 days. On day 5 plaque sizes were measured and plotted. Cumulative data from 4 experiments is shown, each dot represents a single plaque area. B) MEFs were infected for 16 hrs before being fixed and stained for GRA7 to mark the PV. Parasites per vacuole were quantified, counting 100 vacuoles per experiment, and fractions are plotted for each experiment. Cumulative data from 4 experiments is plotted, each dot is fraction obtained from an individual experiment. C) HFFs were infected with 200 parasites per well and allowed various timepoints to attach before being washed extensively and cultured for 5 days before quantifying plaque numbers, which were normalized to the plaque counts from wells that were not washed (=1). Cumulative average +/-SD from 4 experiments is plotted. For panels A-B, statistics performed were one-way ANOVAs with multiple comparisons, and a Holm-Sidak’s correction; **** p < 0.0001 (CEP vs. CEP *Δpigj*), *** p < 0.001 (CEP vs CEP *Δpigj* + *PIGJ* ); ns, not significant. For panel C, t-tests yielded non-significant values.


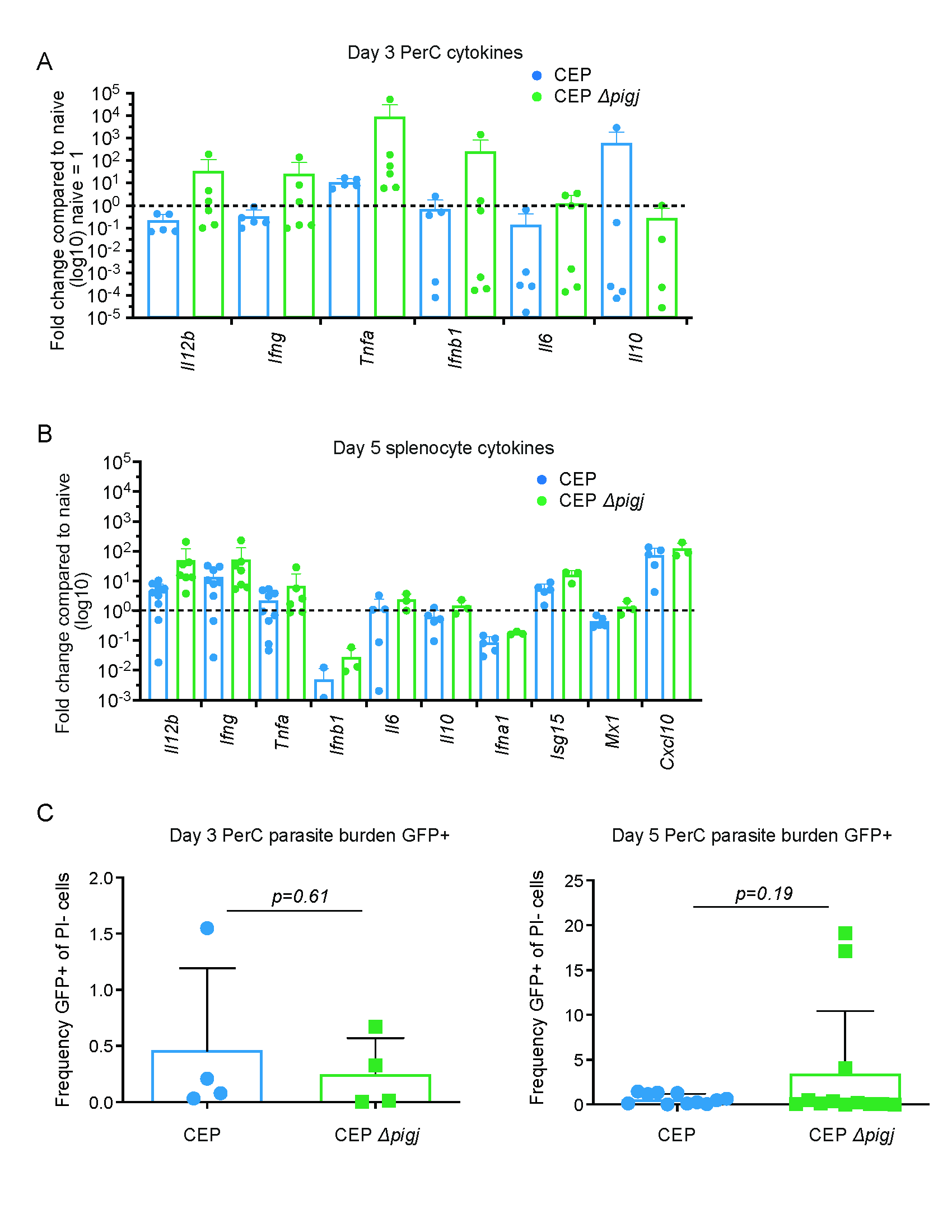


**Figure S10: Similar cytokine response and parasite burden between wildtype and PIGJ mutant strains as revealed by qPCR and flow cytometry.**

A) Peritoneal exudate cells (PerC) on day 3, or B) splenocytes on day 5 were harvested after primary infection with the indicated parasite strains in C57BL/6J mice. RNA was isolated, cDNA synthesized, and qPCR was performed to measure gene expression levels (fold change), which were normalized to uninfected mouse levels (naïve = 1). Cumulative results from 2-5 experiments are plotted. Each dot is the result of an individual mouse. C) Day 3 and 5 days after infection with the indicated GFP expressing parasite strains, PerC’s were harvested, and PI negative cells were analyzed by flow cytometry. Average frequency (+SD) of infected GFP+ PerC among total PI- cells is shown for each infection. Cumulative results from 2-3 experiments are plotted, with each dot representing an individual mouse. Statistics were performed with an unpaired t-test and values indicated that no condition revealed a significant difference between CEP and CEP *Δpigj* infections. This figure complements Figure 6.


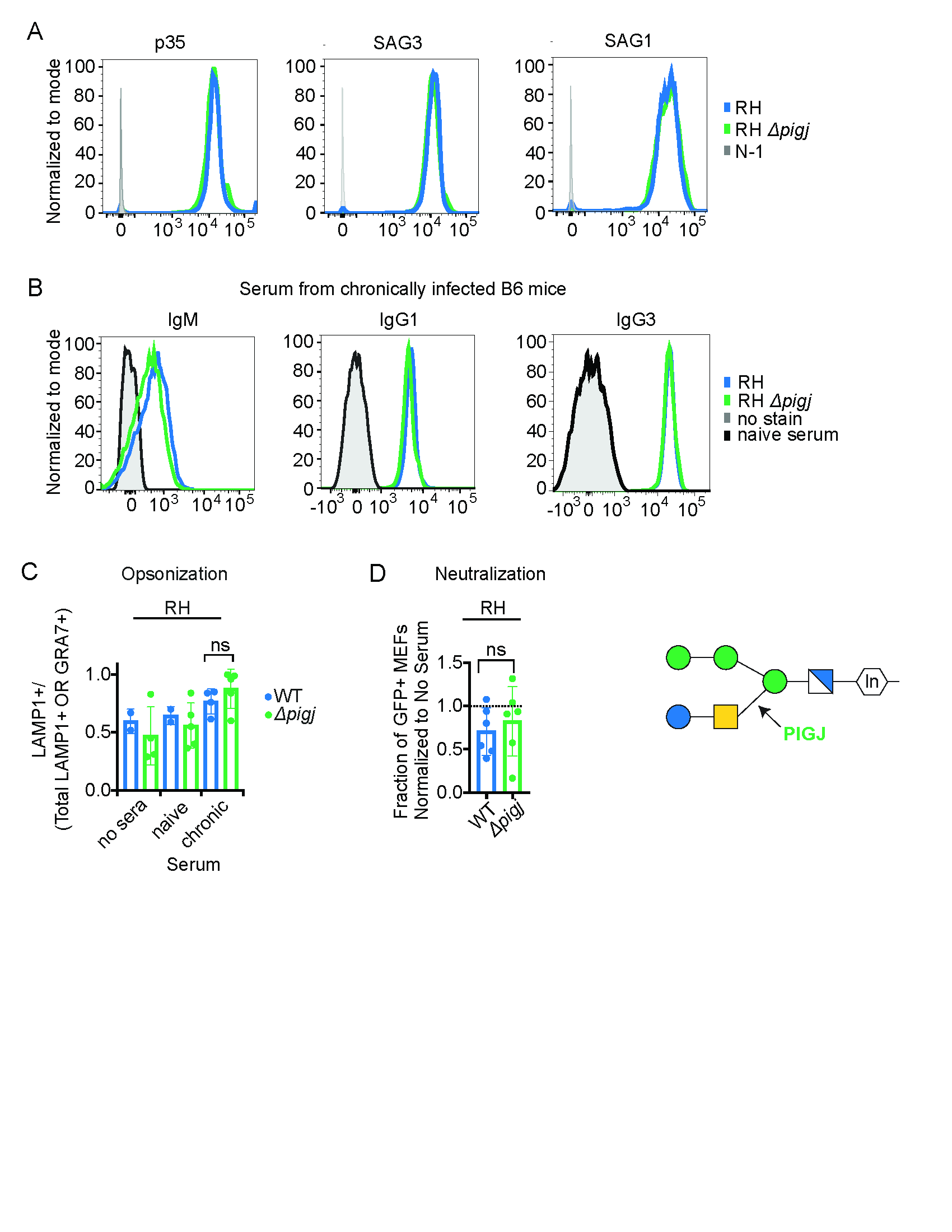


**Figure S11: Surface expression of GPI-anchored SAGs, antibody recognition and functions against RH *Δpigj* strains are intact.**

A) Parasite surface expression of p35, SAG3, and SAG1. Fixed parasites were incubated with primary antibodies against the respective surface antigen, and secondary fluorescent anti-isotype antibodies were used to measure via flow cytometry. Representative histograms of 4 different experiments shown for RH and RH *Δpigj*. B) Fixed parasites were incubated with serum from CEP chronically infected C57BL/6J mice, and antibodies bound to parasites were detected with fluorescent anti-isotype antibodies. Representative histograms of 2-3 experiments displaying parasite-specific antibody reactivity to the indicated RH strains at 10^-2^ serum dilution. C) Parasites were incubated with 1% serum from CEP chronically infected C57BL/6J mice (“chronic serum”) or naïve mice for 20 minutes before allowing to invade or be phagocytosed for 40 minutes. Opsonization was calculated as LAMP1+/ total LAMP1+ or GRA7+ for each parasite observed. Each dot represents the ratio obtained after counting 100 parasites by fluorescence microscopy for an individual serum, and samples were blinded. Plotted is the average ratio +SD; no serum controls were also assessed. D) Parasites were incubated with 10% chronic serum from C57BL/6J mice for 20 minutes before allowing to invade MEFs for 2 hours. MEFs were measured by intracellular staining with FITC-labeled parasite-specific antibodies (RH strains) indicating parasite invasion and normalized to infections in the absence of serum. Each dot represents the result from an individual serum. Statistics for opsonization was calculated with one-way ANOVA with Tukey correction and neutralization were calculated with unpaired t-tests; ns, non-significant. Schematic of the *T. gondii* GPI with the GT activity of PIGJ indicated. This figure complements Figure 7.

**
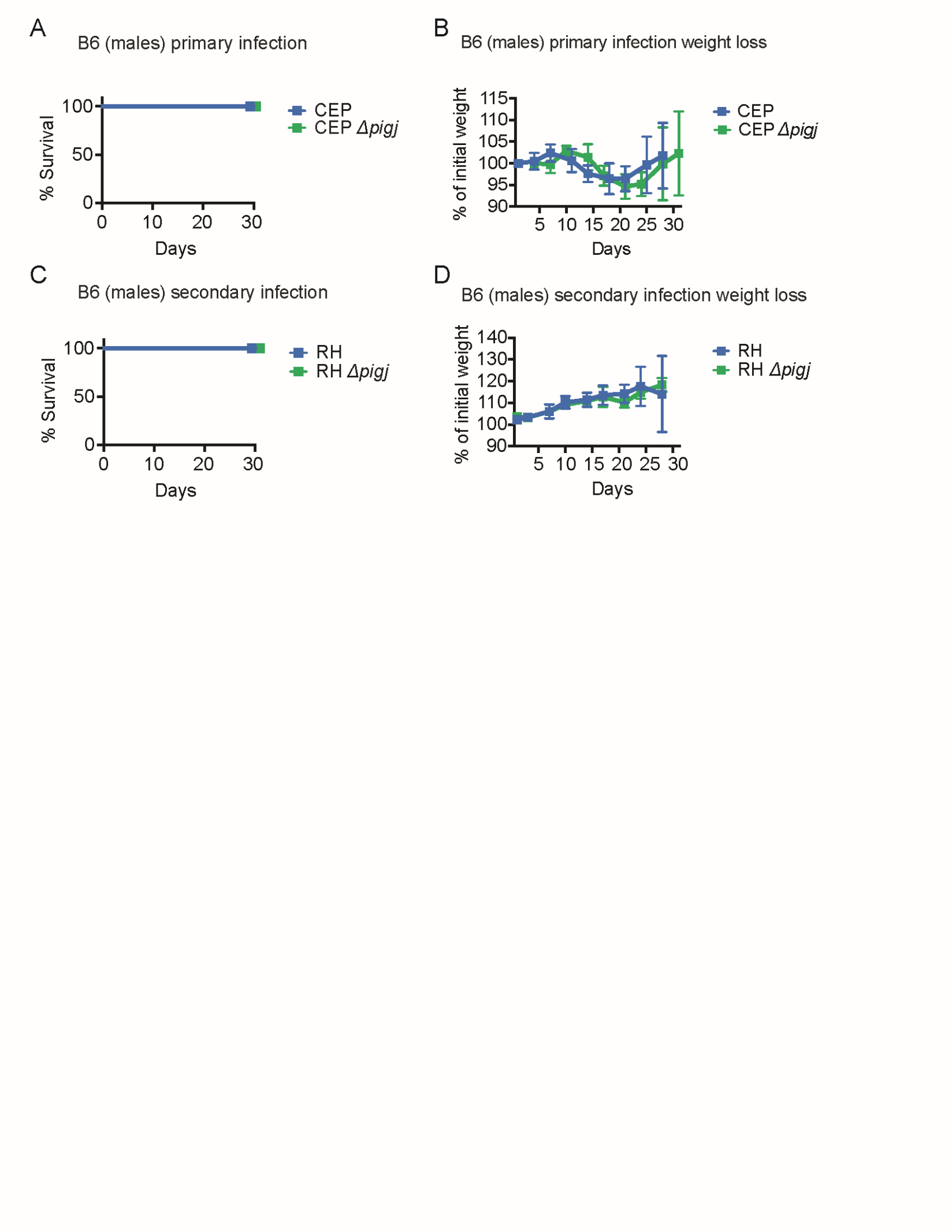
**

**Figure S12: Male C57BL/6J mice are protected against *Δpigj* primary and secondary infections.**

A-B) Male C57BL/6J (B6) mice were given primary infections i.p. with 10^4^ parasites of either CEP or CEP *Δpigj* and monitored for survival in A, and weight loss in B, for 30 days. Cumulative results from 2 experiments are plotted (mice; n = 6 CEP, n = 8 CEP *Δpigj*). C-D) Male B6 mice were first given a primary infection of 10^4^ CEP parasites, and after 35 days were given a challenge infection of either RH or RH *Δpigj* and monitored for survival in C, and average weight loss +/-SD in D. Cumulative results from 2 experiments are plotted (mice; n = 2 RH, n = 4 RH *Δpigj*). For survival analyses, significance was determined by Log-rank (Mantel-Cox) test, and for weight loss, significance was determined by an unpaired t test, none of which were found significant.


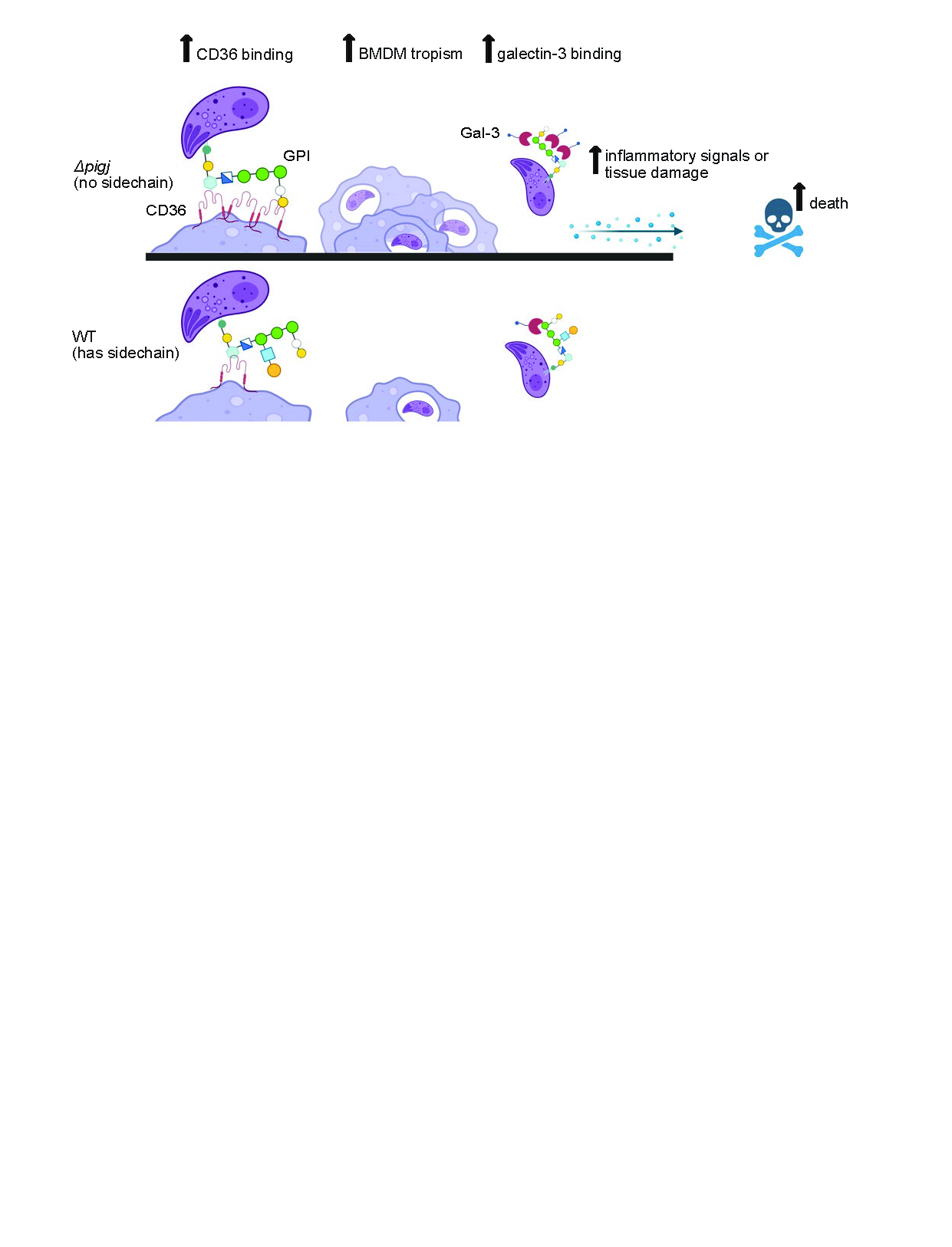


**Figure S13: Working model of *Δpigj* pathogenicity.**

With the loss of the GPI sidechain in PIGJ mutant *T. gondii* strains, we have found an increase in CD36 and galectin-3 parasite binding, and BMDM tropism. We hypothesize there are other inflammatory signals possibly leading to tissue damage that ultimately are responsible for death of the infected hosts, and the cause for increased pathogenicity of GPI sidechain-null parasite strains.
